# COVID-19 and myocarditis: a systematic review and overview of current challenges

**DOI:** 10.1007/s10741-021-10087-9

**Published:** 2021-03-24

**Authors:** Teresa Castiello, Georgios Georgiopoulos, Gherardo Finocchiaro, Monaco Claudia, Andrea Gianatti, Dimitrios Delialis, Alberto Aimo, Sanjay Prasad

**Affiliations:** 1 Department of Cardiology, Croydon Health Service, London, UK; 2 School of Biomedical Engineering and Imaging Sciences, Kings College London, London, UK; 3 The Kennedy Institute of Rheumatology University of Oxford, Oxford, UK; 4 Anatomic Pathology Unit, Papa Giovanni XXIII Hospital, Bergamo, Italy; 5 Department of Clinical Therapeutics, National and Kapodistrian University of Athens School of Medicine, Athens, Greece; 6 Scuola Superiore Sant’Anna, Pisa, Italy; 7 Fondazione Toscana Gabriele Monasterio, Pisa, Italy; 8 Royal Brompton Hospital, Imperial College London, London, UK

**Keywords:** Coronavirus, COVID-19, SARS-CoV-2, 2019nCoV, Myocarditis, Cardiac damage, Cardiac injury, Myocardial damage

## Abstract

Myocardial inflammation in COVID-19 has been documented. Its pathogenesis is not fully elucidated, but the two main theories foresee a direct role of ACE2 receptor and a hyperimmune response, which may also lead to isolated presentation of COVID-19-mediated myocarditis. The frequency and prognostic impact of COVID-19-mediated myocarditis is unknown. This review aims to summarise current evidence on this topic. We performed a systematic review of MEDLINE and Cochrane Library (1/12/19–30/09/20). We also searched clinicaltrials.gov for unpublished studies testing therapies with potential implication for COVID-19-mediated cardiovascular complication. Eligible studies had laboratory confirmed COVID-19 and a clinical and/or histological diagnosis of myocarditis by ESC or WHO/ISFC criteria. Reports of 38 cases were included (26 male patients, 24 aged < 50 years). The first histologically proven case was a virus-negative lymphocytic myocarditis; however, biopsy evidence of myocarditis secondary to SARS-CoV-2 cardiotropism has been recently demonstrated. Histological data was found in 12 cases (8 EMB and 4 autopsies) and CMR was the main imaging modality to confirm a diagnosis of myocarditis (25 patients). There was a substantial variability in biventricular systolic function during the acute episode and in therapeutic regimen used. Five patients died in hospital. Cause-effect relationship between SARS-CoV-2 infection and myocarditis is difficult to demonstrate. However, current evidence demonstrates myocardial inflammation with or without direct cardiomyocyte damage, suggesting different pathophysiology mechanisms responsible of COVID-mediated myocarditis. Established clinical approaches should be pursued until future evidence support different actions. Large multicentre registries are advisable to elucidate further.

**Supplementary information:**

The online version contains supplementary material available at 10.1007/s10741-021-10087-9.

## Background

Coronavirus infection disease 2019 (COVID-19) is a disease caused by severe acute respiratory syndrome coronavirus 2 (SARS-CoV-2), which was initially identified in the city of Wuhan in China in December 2019 and rapidly spread worldwide. The range of clinical presentations includes flu-syndrome symptoms, including cough, fever, fatigue, shortness of breath, anosmia, ageusia, and pharyngodynia which may deteriorate in acute respiratory distress and multiorgan failure. Cardiac involvement with acute myocardial injury is a possible clinical scenario. Previous studies have shown that high troponin levels are associated with increased mortality in patients with COVID-19. However, abnormal troponin levels are not necessarily a sign of acute myocarditis [1, 2]. The aim of this review was to critically summarise current evidence on COVID-19-related myocarditis. In this constantly shifting landscape, we sought to address the many challenges in the early diagnosis and management of myocarditis in patients with COVID-19 infection.

## Methods

This review study was submitted to PROSPERO (CRD189432) and reported according to the Synthesis without meta-analysis in systematic reviews (SWiM) and EQUATOR Reporting Guidelines where applicable [3]. Two independent researchers (TC and GG) performed a systematic review of the MEDLINE and the Cochrane Library for relevant articles in humans, published from 1 December 2019 to 7 January 2021. Eligible studies had laboratory confirmed COVID-19 and a clinical and/or histological diagnosis of myocarditis by the European Society of Cardiology (ESC), World Health Organisation (WHO, International Society and Federation of Cardiology (ISFC) criteria.

Details of search strategy, eligibility, data extraction and synthesis and heterogeneity in reported effects, quality assessment and certainty of evidence are provided in the Supplementary File.

## Results

From 490 initial records, we screened as 353 full-text articles (Supplementary Fig. 1). After further excluding 61 articles (Supplementary File, reference list), 38 case reports in patients with confirmed COVID-19 were compatible with a definite or probable diagnosis of acute myocarditis by ESC or WHO/ISFC criteria. These cases are presented in detail in Supplementary Table 2.

**Fig. 1:**
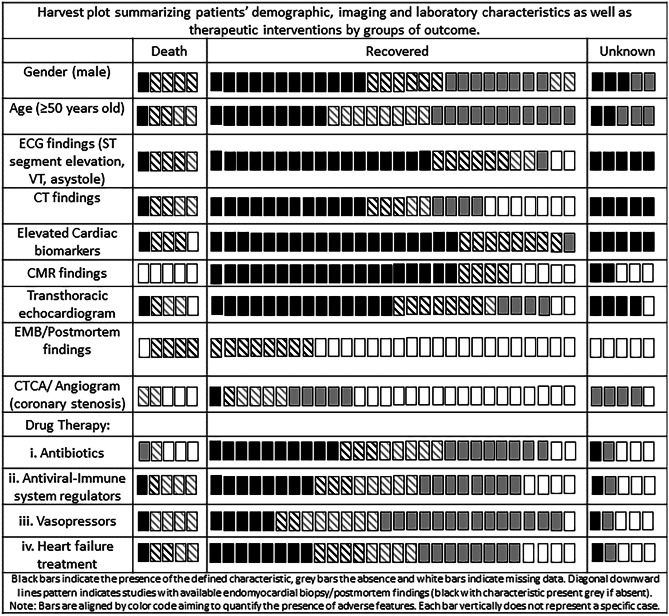
Harvest plot summarizing demographic characteristics, imaging findings and treatment in patients with COVID-19 and myocarditis to the outcome

Table 1 displays the main clinical characteristics, the diagnostic workup and outcome of eligible cases, whereas a harvest plot (Fig. 1) was implemented to synthesize the evidence on the prevalence of high-risk features (ECG, biomarkers, CMR, etc.) according to the outcome (death, recovery, undetermined) in aggregated cases [4].

**Table 1:** Characteristics of cases published with possible or definite diagnosis of myocarditis and COVID-19

	Clinical	Origin/race	Hx	Blood markers	ECG	CXR/CT	CTCA/CA	ECHO	CMR	EMB/PM	ESC criteria^18,*^	Outcome
Hu et al. [suppl 8]	Chest pain, dyspnoea	37F, China	N/A	+	+	+	N	+	N/A	N/A	3	Recovered
Paul et al. [suppl 9]	Chest pain, fatigue	35 M, French	obesity	+	+	N	N/A	+	+	N/A	4	Recovered
Coyle et al. [suppl 10]	Upper respiratory, dyspnoea and gastrointestinal	57 M, USA	HTN	+	+	+	N	+	+	N/A	4	Recovered
Kim et al. [suppl 11]	Fever, cough, diarrhoea, dyspnoea	21F, Korean	N/A	+	+	+	+	+	+	N/A	4	N/A
Craver et al. [suppl 12]	Dizziness, headaches, collapsed	17 M, African American	N/A	N/A	+	N/A	N/A	N/A	N/A	+ (PM)	1	Death
Inciardi et al. [suppl 3]	Fatigue, fever, cough	53F, Italian	Clear	+	+	N	N	+	+	N/A	4	Recovered
Sala et al. [suppl 4]	Chest pain, dyspnoea	43F, Italian	Clear	+	+	+	+	+	+	+	4	Recovered
Doyen et al. [suppl 5]	Fever, cough, gastrointestinal	69 M, Italian	HTN	+	+	+	N	N	+	N/A	4	Recovered
Varga et al. [suppl 6]	Dyspnoea, fever, tachycardia, confusion	71 M, N/A	Renal transplant, CAD, HTN	+	+	+	N/A	N	N/A	+ (PM)	1	Death
Luetkens et al. [suppl 7]	Fatigue, dyspnoea, syncope	79 M, Germany	asthma	+	N	+	N/A	N	+	N/A	3	Recovered
Trogen et al. [suppl 13]	Gastrointestinal, neck pain	17 M, USA	Obesity, asthma	+	+	+	N/A	+	+	N/A	4	Recovered
Besler et al. [suppl 14]	Febrile sensation and chest pain	20 M, Turkish	Clear	+	N/A	+	N/A	N/A	+	N/A	3	Recovered
Gnecchi et al. [suppl 15]	Chest pain, fever	16 M, Italian	Clear	+	+	N	N/A	+	+	N/A	4	Recovered
Garot et al. [suppl 14]	Cough, fever, fatigue	18 M, French	Clear	+	+	+	N/A	+	+	N/A	4	Recovered
Al-Assaf et al. [suppl 17]	Asymptomatic	58 M, Dubai	HTN	N	N	N	+	N	+	N/A	2	Recovered
Salamanca et al. [suppl 18]	Dyspnoea, syncope	44 M, Spanish	N/A	+	+	+	N	+	+	+	4	Recovered
Bonnet et al. [suppl 19]	Respiratory distress	27 M, French	Clear	+	+	N/A	N/A	+	+	N/A	4	Recovered
De Vita et al. [suppl 20]	Fatigue, dyspnoea, orthopnea	35F, Italian	Childbirth one month ago	+	+	+	N/A	+	+	N/A	4	Recovered
Pavon et al. [suppl 21]	Fever, cough	64 M, Switzerland	Pulm. sarcoidosis, Epilepsy	+	N	+	N	N	+	N/A	3	Recovered
Ford et al. [suppl 22]	Malaise, fever, chest pain	53 M, USA	Dyslipedimia	+ ^**^	+	+	N/A	+	+	N/A	4	Recovered
Richard et al. [suppl 23]	Lethargic, coffee ground emesis	28F, Caucasian	T1DM, asthma, depression	+	+	+	N	+	+	N/A	4	Recovered
Warchol et al. [suppl 24]	VT with signs of instability	74 M, Poland	T2DM, Afib, HTN	+	+	N/A	N/A	N/A	+	N/A	4	Recovered
Jacobs et al. [suppl 25]	Diarrhoea, cough, dyspnoea	48 M, Belgium	HTN	+	+	+	N/A	N	N/A	+ (PM)	3	Death
Hua et al. [suppl 26]	Chest pain, dyspnoea, cough	47 M, Afro-Caribbean	Clear	+	+	+	N	+	N/A	N/A	3	N/A
Cizgici et al. [suppl 27]	Chest pain, dyspnoea	78 M, Turkish	N/A	+	+	+	N	N/A	N/A	N/A	2	N/A
Hussain et al. [suppl 28]	Fever, dyspnoea, gastrointestinal	51 M, Italian	HTN	+	+	+	N	+	N/A	N/A	3	N/A
Spano et al. [suppl 29]	Dyspnoea, orthopnea, weakness	49 M, Switzerland	Clear	+	+	+	N/A	+	+	N/A	4	N/A
Dalen et al. [suppl 30]	Near syncope, chest discomfort	55F, Norway	Clear	+	+	N, N/A	N/A	+	+	N/A	4	Recovered
Labani et al. [suppl 31]	Flu-like symptoms, chest pain	71F, French	Breast cancer	+	+	+	N/A	+	+	N/A	4	Recovered
Khatri et al. [suppl 32]	Fever, malaise, dyspnoea	50 M, USA	HTN, Stroke	+	+	+	N	+	N/A	N/A	3	Death
Albert et al. [suppl 34]	Fever, dyspnoea	49 M, USA	Smoking	+	+	N	N/A	+	N/A	+	3	Recovered
Nicol et al. [suppl 35]	Fever, odynophagia, neck pain	40 M, France	Obesity	+	+	+	N	+	+	+	4	Recovered
Gauchotte et al. [suppl 35]	Fever, fatigue, abdominal pain	69 M, France	T2DM, HTN, IHD	+	+	N	N	+	N/A	+ (PM)	3	Death
Cafarra et al. [suppl 36]	Dyspnoea, palpitations	40, F, Italy	CKD, lymphocytic myocarditis	+	+	N/A	N	+	N/A	+	3	Recovered
Iqbal et al. [suppl 37]	Orthopnea, dyspnoea, cough	40 M, USA	T2DM	+	+	N/A	N	+	+	N/A	4	Recovered
Othenin et al. [suppl 38]	Myalgia, abdominal pain, fever	22 M, Easr Africa	N/A	+	N	+	N/A	+	N/A	+	2	Recovered
Escher et al. [suppl 39]	Fever, dyspnoea	48 M, Germany	N/A	+	N/A	N/A	N	+	N/A	+	2	Recovered
Escher et al. [suppl 39]	Headache, fever	39 M, Germany	N/A	+	+	N/A	N	+	+	+	3	Recovered

Italicized symptoms indicate clinical presentations suggestive of myocarditis. Blood markers: N-terminal prohormone of brain natriuretic peptide, troponin I or T, C-reactive protein, creatine kinase myocardial band

^*^ESC criteria indicates the number of fulfilled ESC Diagnostic Criteria for myocarditis: (I) ECG/Holter/stress test, (II) myocardiocytolysis markers (troponin I or T), (III) functional and structural abnormalities on cardiac imaging (esco/angiography/CMR), (IV) tissue characterisation by CMR. Symbol “+” indicates the presence of a pathological characteristic

^**^Elevated brain natriuretic peptide with normal troponin

*N/A* not available, *N* normal, *Μ* male, *F* female, *Hx* medical history, *ECG* electrocardiogram, *CXR* chest X-ray, *CT* computed tomography, *CTCA/CA* computed tomography of coronary angiography/coronary angiography, *ECHO* transthoracic echocardiography, *EMB* endomyocardial biopsy, *DM* diabetes mellitus, *T1DM* type 1 DM, *T2DM* type 2 DM, *HTN* hypertension, *CKD* chronic kidney disease

### Clinical presentation

About 45% of the cases had fever (*n* = 17) at presentation or mild temperature increase, while no significant abnormal findings in lung imaging were reported in 9 patients. Eight patients had gastrointestinal symptoms, and 4 patients had previous or presenting syncope.

### Laboratory findings

Troponin levels were available in 36 out of 38 cases and varied substantially. BNP was raised in 22 cases (Table 1).

### Electrocardiogram

Electrocardiogram was normal at presentation in four patients only and electrocardiographic finding differed quite broadly (Fig. 1).

### Echocardiography

Out of 34 patients with available echocardiographic data, in seven cases, there was no any structural or functional abnormality (Table 1).

### Cardiac magnetic resonance

Two thirds of the patients (25 out of 38) were tested with cardiac CMR with late gadolinium enhancement (LGE) evident in one half. Diffuse oedema and myocardial inflammation were captured by dedicated sequences in 19 cases (Table 1).

### Biopsy and histology

EMB was performed only in 8 cases; however, only one case report clearly described presence of SARS-CoV-2 in the cardiomyocyte [suppl 33]. Histology demonstrated inflammation of the myocardium with predominance of macrophages, and myocyte necrosis was instead limited. The case presented clinically with acute myocarditis with cardiogenic shock, and EMB results suggests a viral mediated inflammation without significant direct myocyte destruction. Histology evidence of SARS-CoV-2 were also found in 5 out of 104 EBM with suspected myocarditis or heart failure; however, only 2 met Dallas criteria of myocarditis [suppl 15, 38]. Histological data from autopsy were also available in 4 patients and included accumulation of inflammatory cells in the endothelium [suppl 6], inflammatory infiltrates [suppl 12], and signs of ferroptosis [suppl 25].

### Treatment

Therapeutic regimens varied significantly. Ten patients were treated with hydroxychloroquine, 3 received lopinavir/ritonavir and 4 tocilizumab. Antibiotics were administered in 14 patients, steroids in 13 and heart failure medications in 14 cases. Eight patients received anticoagulants. An aldose reductase inhibitor (for research purposes), cyclophosphamide and azathioprine were administered in one case each.

### Outcome

**Fig. 2:**
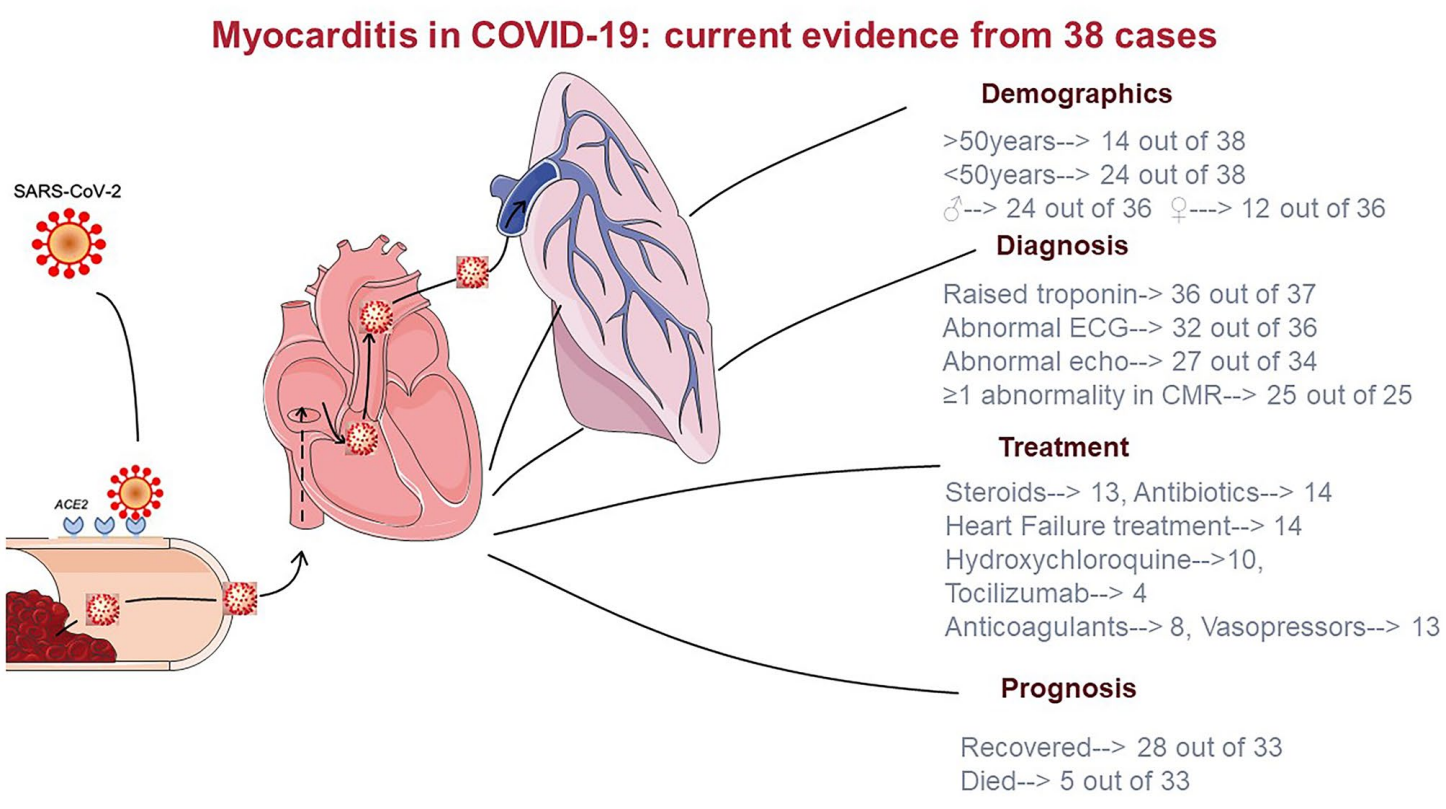
Central illustration. Current evidence on acute myocarditis in patients with COVID-19

Overall, 28 patients recovered and were discharged from hospital while 5 patients died. Five case reports did not specify the outcome (Table 1). A harvest plot summarising available evidence on COVID19 cases with probable or definite myocarditis is presented based on the availability of histological data is presented in Fig. 1 and a summary of the demographic, pathological diagnostic findings and drugs categories used is presented in Fig. 2.

### Overview of findings from the literature

To date, there is an extreme paucity of published data on COVID-19-related myocarditis. In our review study and despite the systematic search, we retrieved only case reports of this entity with substantial heterogeneity with respect to demographics and clinical presentation as well as imaging and histologic findings. Building on this sparse evidence, we discuss pathophysiological mechanism, and challenges in the diagnosis and management of COVID-19-related myocarditis.

## Discussion

### Epidemiology of COVID-19-related myocarditis

While the incidence of myocarditis is estimated as 22 per 100,000 [5], the true epidemiology of COVID-19 myocarditis is difficult to establish. This review only summarises reported cases and does not reflect the true incidence in COVID-19, which, for several reasons, is likely to be higher. In fact, the cases presented are at the more severe end of the spectrum of cardiac manifestation, and in hospitalised patients, the severity of the respiratory status may have masked a cardiac involvement. We may add that access to cardiac tests was limited due to safety concerns.

Epidemiological information is more robust with respect to cardiac injury, defined as evidence of elevated cardiac troponin values with at least one value above the 99th percentile upper reference limit (URL). Cardiac injury has been reported in 19–28% of patients diagnosed with COVID-19 [6–8].

Among the confirmed deaths reported by the National Health Commission of China, the 11.8% of deceased people exhibited raised troponin I or had a cardiac arrest during hospitalisation [9]. Similarly, a report on 150 patients from Wuhan showed that 33% of COVID-19 deaths were also attributable to cardiac disease with an additional 7% of death ascribed to cardiac damage alone [10].

### Myocardial injury vs acute myocarditis

Raised troponin is often observed in myocarditis, but differentiation between myocardial injury and myocarditis may be challenging [11]. In the absence of myocardial ischaemia, a significant troponin rise may be indicative of acute myocarditis in the appropriate clinical context. Under normal circumstances, a series of cardiac investigations would follow the detection of raised troponin blood levels, when acute myocarditis is suspected. As previously discussed, the limited availability of second level testing (including CMR to ascertain an acute myocarditis in the context of COVID-19 infection) for COVID-19 suspected myocarditis means that often a final diagnosis cannot be made. Relying on relatively basic imaging tests such as echocardiography often results in a diagnostic conundrum where the aetiological mechanisms underlying clinical manifestations are often unclear.

### Immunological and pathophysiological mechanisms

The pathogenesis of acute myocardial damage in COVID-19 is currently unknown. The two main theories foresee a direct role of angiotensin-converting enzyme 2 receptors (ACE2) and a hyperimmune response. SARS-CoV-2 binds to the ACE2 via its spike protein and enters various cells, including epithelial cell and macrophages. Receptors binding SARS-CoV-2 are also expressed in the myocardium, and murine models showed that the virus can regulate ACE2 activity, and indirectly causing myocardial inflammation [12].

Although COVID‐19 RNA has been found in the interstitial myocardium [suppl 6, 26] and SARS‐CoV‐2 gene specific sequences were found in EMBs [13], the exact mechanisms of myocardial injury in SARS-CoV-2 infection remain still largely unknown. Escher et al*. *[suppl 39] detected SARS‐CoV‐2 genome in 5 of 104 EMB of patients with suspected myocarditis or unexplained heart failure. However, only two patients met Dallas criteria for myocarditis. On the remaining patients, authors suggested alternative histopathological mechanism of myocardial damage, such as vascular involvement with arterial obliteration leading to ischaemia [13]. The first published case of EBM-proven case, which met the Dallas criteria for myocarditis, was a virus-negative lymphocytic myocarditis [suppl 4], consistent with an immune-mediated pathogenesis. In this well-accepted hypothesis, the virus triggers a cascade of hyperinflammatory response, which leads to cardiac damage. Based on current limited evidence, the pathophysiological mechanism can be explained either by immune response related to the cytokine storm or by intrinsic process commenced in the myocardium.

The first case report of COVID-19-mediated myocarditis corroborated by histological evidence of direct SARC-CoV-2 cardiomyocyte infection [suppl 35] was associated to limited myocyte necrosis supporting the hypothesis of hyperinflammatory response involving the myocardium, rather than direct cardiomyocyte loss for viral infection.

In 4 additional cases with COVID-19 and a clinical presentation compatible with acute myocarditis [suppl 6, 12, 18], postmortem histological examination of the heart showed diffuse inflammatory infiltrates and foci of myocardial necrosis. An abnormal immune response characterised by an increased number of neutrophils and monocytes, reduced lymphocyte populations and elevated systemic pro-inflammatory cytokines and growth factors, has indeed been associated with severe COVID-19 [14–16] [suppl 4]. It should be acknowledged that the postmortem diagnosis of myocarditis should include toxicology and molecular investigation beyond standardised morphologic criteria and histological sampling [17]; this information was not available in all published cases.

Cardiac injury more often coexists with severe COVID19 and multiorgan failure, while myocarditis remains anecdotal. In order to differentiate myocarditis from cardiac injury in the context of severe COVID-19 and/or multiorgan failure, we employed the diagnostic criteria for acute myocarditis proposed by the ESC, requiring at least a compatible clinical presentation and one diagnostic criterion along with the exclusion of coronary artery disease (angiographically) and other cardiovascular or non-cardiac causes [18]. Cardiogenic shock may mimic fulminant myocarditis, in the absence of histological evidence of myocarditis [suppl 26]. The possible mechanisms of cardiac damage leading to cardiogenic shock are a mismatch between oxygen supply and demand, respiratory acidosis and hypoxia, and excessive intracellular calcium causing cardiac myocyte apoptosis. Although the pathogenesis of different cardiac injury may be similar and related to the cytokine storm triggered by the host immune response, the specific cardiac damage may vary.

### Diagnostic red-flags for COVID-19-related myocarditis

Several laboratory findings may raise the suspicion of COVID-19 infection in the appropriate clinical context. Lymphocytopenia is often detected (in up to 83%), while leukopenia and thrombocytopenia can occur in about a third of cases. Significant elevation of inflammatory markers (D-dimer, ferritin, and C-reactive protein) is observed in more aggressive forms [19]. Troponin rise correlates with a hyperinflammatory state, is a sign of myocardial injury and may indicate acute myocarditis. Elevated troponin in the absence of other laboratory markers suggestive of aggressive COVID-19 disease should direct towards an isolated cardiac presentation. On the contrary, high concentrations of troponin combined with generalised increase of inflammatory markers suggests multiorgan failure and a hyperinflammatory response [20, 21]. Similarly, NT-proBNP is associated with poor outcomes and reflects haemodynamic overload and dysfunction.

Electrocardiographic changes in myocarditis are not pathognomonic, since a variety of ECG patterns from sinus tachycardia and ectopic beats to ST elevation and T-wave inversion have been described.

Transthoracic echocardiography (TTE) is the first-line imaging test performed in patients with a suspicion of acute myocarditis. Echocardiography findings in COVID-19-related myocarditis included both global and regional hypokinesia [suppl 3-5, 8, 11, 15, 20, 28, 29], as well as an increase in wall thickness, which is suggestive of myocardial oedema, although hypertension, with subsequent LVH, is a common preexisting cardiovascular condition [suppl 4]. Yet, seven patients with COVID-19 and probable myocarditis had a normal echocardiographic exam [suppl 5-7.9,17,19,25].

CMR enables tissue characterization and can lead to a diagnosis of myocarditis according to Lake Louise criteria [22].

In COVID-19, reported cases have shown increased T2 values and positive short T1 inversion recovery (STIR) typical for myocarditis [suppl 3,4,7,10,13]. Sub-epicardial LGE was described in two cases [suppl 5,7], extensive transmural LGE was present in one case [suppl 11], while diffuse LGE involving the entire biventricular wall was seen in a 53-year-old woman with isolated cardiac involvement [suppl 1]. In the first biopsy proven case, myocardial oedema was detected, but late gadolinium enhancement was absent [suppl 2]. Pathological diagnostic features are presented in total in Fig. 2.

EMB remains the gold standard for the diagnosis of myocarditis, but limitations of the technique need to be taken into account for cases that are not biopsy proven [23]. The most common histopathology type is lymphocytic myocarditis, typically characterised by infiltrates of T lymphocytes and macrophages, with sparse B-lymphocytes [24]. The first EMB-proven COVID-19 myocarditis was consistent with lymphocytic myocarditis, with evidence of T-lymphocytic inflammatory infiltrates and significant interstitial oedema [suppl 2]. Instead, the first described clinical presentation suggestive of fulminant myocarditis in the context of COVID-19 septic shock did not meet Dallas criteria for myocarditis [suppl 26]. This discrepancy has been already noted with other viral infection [25]. Figure 1 summarises the characteristics of cases based on whether histological data were available.

### Findings from postmortem examination

According to the Association for European Cardiovascular Pathology guidelines for the diagnosis of myocarditis [17], a patchy inflammatory infiltrate is not sufficient to diagnose myocarditis in the absence of myocyte necrosis. In lymphocytic myocarditis, for instance, polymerase chain reaction (PCR) on blood and myocardium is the gold standard to diagnose myocarditis [17].

**Fig. 3:**
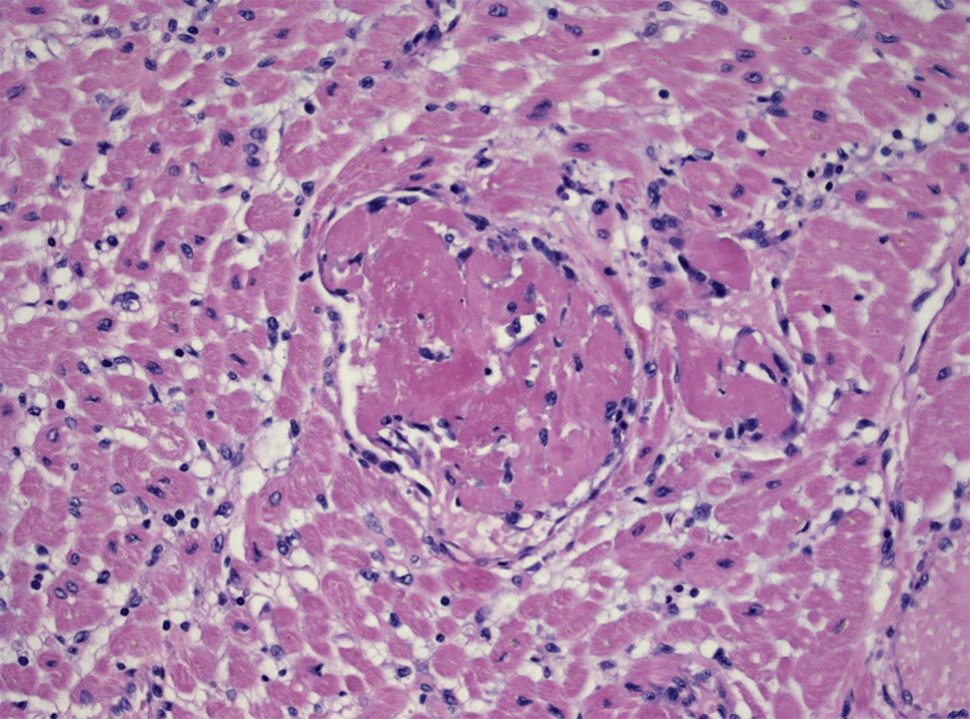
Microscopic study of the heart from a deceased patient with COVID-19 and cardiogenic shock in Bergamo, Italy. Numerous microthrombi of the left ventricle are shown without evidence of an inflammatory infiltrate; the detection on tissue by molecular technique for SARS-CoV-2 was negative. Collectively, the diagnosis of COVID-19 related myocarditis was excluded in view of the thorough histological evaluation that proved absence of myocyte necrosis and inflammatory infiltrate

An international multicentre group [26] performed 21 autopsies of individuals deceased from COVID-19. In 3 cases, the diagnosis of lymphocytic myocarditis was made, while in 18 cases (86%), there was widespread macrophage infiltration in the myocardium, without myocyte injury. Similarly, in 2 EMBs reported by a German study [27], myocardial inflammation with increased lymphocytes and macrophages was documented. Interestingly, patients were tested negative for COVID-19 in nasopharyngeal swab; however, IgG antibodies were positive and, most significantly, SARS-CoV-2-specific nucleic acids, performed by an RT-PCR assay, were detected in the myocardium, suggesting that the myocarditis presentation does also occur in healed respiratory presentation or otherwise paucisymptomatic COVID-19. Evidence of SARS-CoV-2 mRNA in the myocardium has been also found in 5 out of 12 COVID-19 autopsies, although myocarditis was not diagnosed [28]. Accordingly, in an unpublished case (detailed description in the Supplementary File), the extensive microscopic study of the heart showed numerous microthrombi of the LV without evidence of an inflammatory infiltrate; tissue detection for SARS-CoV-2 by molecular technique was negative (Fig. 3).

A recent report of the National Health Commission of the People’s Republic of China has confirmed the findings of myocyte necrosis and mononuclear cell infiltrates in the myocardium (http://kjfy.meetingchina.org/msite/news/show/cn/3337.html (7th edition).

### Prognosis

Raised troponin levels in COVID-19 are associated with worse outcome [suppl 7], but the specific prognostic role of myocarditis is unknown [9, 29]. A retrospective single-centre study conducted in Wuhan showed significant correlation between myocardial injury and mortality [19] and worse outcome even when compared with patients with normal troponin levels and preexisting cardiovascular disease. While inflammation was described as a possible cause of myocardial injury, myocarditis was not proven in these patients [7]. Myocarditis can present even after resolution of the upper respiratory tract infection or aggravate SARS-CoV-2 infection [suppl 5, 40, 41]. The occurrence of myocardial injury appears to be independent of preexisting cardiovascular risks. Ventricular dysfunction is associated to higher COVID-19 mortality but whether past medical history of old myocarditis with preserved function is associated with worse outcome or predisposes to myocardial damage in COVID-19 is unknown. Our systematic review showed that acute myocarditis in COVID-19 has a moderate prognosis if promptly treated, since only three fatalities were reported while a fourth presented with cardiac arrest and no treatment was administered. Nevertheless, given the potential underreporting of relevant cases, only preliminary conclusions can be drawn.

### Therapy and ongoing trials

At present, treatment for viral myocarditis is largely supportive, including mechanical circulatory support for critical patients. When LV systolic dysfunction occurs, heart failure therapy by well-established guidelines is recommenced in a patient with sufficient cardiac output and haemodynamic stability. There is a current consensus in treating myocarditis patients with biopsy‐proven viral clearance by PCR with immunosuppressive agents such as prednisone and azathioprine, but this may not be necessarily applicable to COVID-19, as the pathophysiology is not fully known [30].

Ten of the cases reported in literature were both empirically treated with hydroxychloroquine and/or antiviral drugs and 12 with corticosteroid as per local protocols while only four patients to date were treated with tocilizumab [suppl 10, 18, 35, 38] (Fig. 1). An additional case presented as acute coronary syndrome and was initially treated with antiplatelets and fondaparinux. When acute coronary disease was excluded by coronary angiogram and CMR showed myocardial oedema and subepicardial enhancement, hydrocortisone was given at day 11 for 9 days [suppl 42]. Ιn total, 14 cases in literature received treatment for heart failure [suppl 3, 4, 7, 9, 10, 39] (Figs. 1 and 2).

Timing of discharge for this subgroup of patients with COVID-19 should follow local protocols for COVID-19 infection resolution. This may vary broadly; however, we would assume that discharge readiness from COVID-19-mediated myocarditis do not differ from myocarditis with other aetiologies. Myocarditis patients should in fact have normalised or decreasing troponin levels with no evidence of malignant arrhythmias on ECG monitoring. In the presence of systolic dysfunction, heart failure therapy should be initiated prior to discharge and close follow-up should be offered. Of note, 28 out of the 38 cases (and out of the 33 cases with available outcome) in our systematic review were discharged from hospital with recovered cardiac function.

Current trials are exploring immunosuppressant therapy for the hyperinflammatory phase that may be beneficial for COVID-19-related myocarditis. Despite none of the trials is specifically designed for myocarditis, future subgroup analyses may identify promising treatments. Some of the ongoing trials are summarised in Supplementary Table 3.

### Study limitations

Inference of our systematic review and generalisability of our findings are significantly hampered by the lack of available studies on COVID-19-related myocarditis. The data of case reports were also incomplete, leading to the exclusion of cases for incomplete interpretation of results. Histologic analysis was limited too, and although ESC criteria and CMR evidence were used to support the diagnosis, the risk of biased information or data interpretation remains elevated. In this context, the synthesis of evidence was qualitative, and no assessment of publication and other form of bias could be performed. Therefore, certainty of evidence is low. This study presents an interim assessment of current data while we are waiting for results from large, multicentre registries.

## Conclusions

Myocardial involvement in COVID-19 is associated with a worse prognosis, but isolated myocarditis is not necessarily a marker of poor prognosis (Fig. 2). However, given the paucity of published data and the inhomogeneity of the cases, conclusive assertion on prognosis cannot be made. Immunosuppressants are under study and have a rationale in systemic hyperinflammation syndrome involving the heart, but data are limited. RECOVERY trial discourages the use of dexamethasone in patient not requiring oxygen; however, dedicated sub-studies on myocarditis have not been made. Colchicine in now under investigation, but again, specific effect on myocarditis is only a supposition [31]. Despite in many cases is not possible to demonstrate cause-effect relationship between SARS-CoV-2 infection and myocarditis, the most recent histology data report direct viral infection of the cardiomyocyte. These evidence of myocarditis with and without direct myocyte damage suggests different pathophysiology mechanisms responsible of COVID-mediated myocarditis and open to diverse therapeutic approaches. The presence or absence of virus in the myocytes identified by EBM is in fact decisive to diagnose correctly COVID-19 myocarditis and to identify the most adequate therapy, since virus-negative patients are more likely to benefit from immunosuppressant therapy, while viral presence in the myocytes may respond to antiviral drugs [7, 29]. Established clinical approaches should be pursued until future evidence support different actions. Large multicentre registries are advisable to elucidate further.

## Supplementary information

Below is the link to the electronic supplementary material.

Supplementary file1 (DOCX 294 KB)

## Abbreviations

(COVID-19): Coronavirus infection disease
(ESC): European Society of Cardiology
(WHO): World Health Organisation
(ISFC): International Society and Federation of Cardiology
(CMR): Cardiac magnetic resonance
(EMB): Endomyocardial biopsy
(ACE2): Angiotensin-converting enzyme 2
(SARS-CoV-2): Severe acute respiratory syndrome coronavirus 2
