# Supplementary material for: COVID-19 and myocarditis: a systematic review and overview of current challenges
Source: Heart Fail Rev. 2021 Mar 24;27(1):251–61. doi: 10.1007/s10741-021-10087-9 (PMC7988375; doi:10.1007/s10741-021-10087-9)
Supplement: Supplementary file 1 — Supplementary file1 (DOCX 294 KB) [file 10741_2021_10087_MOESM1_ESM.docx]

**COVID-19 and Myocarditis: A Systematic Review and Evaluation of Current Challenges**

Teresa Castiello^1,2*^, Georgios Georgiopoulos^2*^, Gherardo Finocchiaro^2^, Monaco Claudia^3^, Andrea Gianatti^4^, Dimitrios Delialis^5^, Alberto Aimo^6,7^, Sanjay Prasad^8^

*^1^ Department of Cardiology, Croydon Health Service, London*

*^2^ School of Biomedical Engineering and Imaging Sciences, Kings College London, London*

*^3^ The Kennedy Institute of Rheumatology University of Oxford, Oxford*

*^4^ Anatomic pathology unit, Papa Giovanni XXIII Hospital, Bergamo.*

^5^ *Department of Clinical Therapeutics, National and Kapodistrian University of Athens School of Medicine, Athens, Greece*

*^6^ Scuola Superiore Sant’Anna, Pisa, Italy;*

*^7^ Fondazione Toscana Gabriele Monasterio, Pisa, Italy;*

*^8^ Royal Brompton Hospital, Imperial College London, London*

**SUPPLEMENTAL MATERIAL**

1. **Search strategy**
2. **Data extraction and synthesis**
3. **List of references of excluded articles**
4. **Unpublished case-report**
5. **Supplementary Tables**
6. **Supplementary Figure**
7. **Search Strategy**

The following keywords and medical subject heading (MeSH) terms were used in our search strategy: “Covid”, “Coronavirus”, “COVID-19”, “SARS-CoV-2”, “2019nCoV” AND “myocarditis”, “cardiac damage” or “cardiac injury” or “myocardial damage”. Articles were limited to those published in the English language. A manual search for references from eligible articles as well as from published review articles was also employed to identify additional relevant studies. Title and abstract were screened at the initial search, followed by full texts of relevant articles where applicable. Duplicate cohorts were excluded.

The following algorithm was used for search in Pubmed:

“Covid-19” OR “novel coronavirus” OR “2019nCoV” OR “novel coronavirus 2019” OR “SARS-CoV-2” OR “Covid” OR “Coronavirus” AND “myocarditis” OR “cardiac damage” OR “cardiac injury”. Individual results for search combinations are provided in Table 1S.

*Eligibility*

We reviewed case reports, case series and cohort studies (including case control and retrospective studies) that assessed patients with laboratory confirmed COVID-19 (either based on RT-PCR or antibody testing) and a detailed work-up aiming to establish the diagnosis of acute myocarditis by the diagnostic criteria for clinically suspected myocarditis according to the European Society of Cardiology (ESC) ^1^ or histological, immunological and immunohistochemical criteria of WHO / International Society and Federation Cardiology (ISFC) criteria. In specific, myocarditis was suspected if there was at least one clinical presentation and at least one diagnostic criterion in the absence of angiographically detectable coronary artery disease and known pre-existing CVD or other extra-cardiac cause, as previously described ^1^. In case of an asymptomatic patient, at least two diagnostic criteria are required for clinically suspected myocarditis.^1^. In the absence of formal fulfilment of the DALLAS criteria ^1^ at endomyocardial biopsy (EMB) or autopsy, imaging methods, especially dedicated Cardiac Magnetic Resonance (CMR) sequences, were considered diagnostic of acute myocarditis when compatible with the Lake Louise Criteria ^2^. Studies with post-mortem findings consistent with acute myocarditis were also included. In contrast, studies reporting acute cardiac injury or myocardial damage without corroborating evidence of myocarditis were deemed ineligible.

*Heterogeneity in reported effects, quality assessment and certainty of evidence*

In view of the results of our systematic search (limited to case reports), we did not employ standardised tools to quantify heterogeneity among reported effects and to evaluate the quality of studies. Respectively, the quality of evidence was classified at the lowest scale of the Quality Rating Scheme according to the Oxford Centre for Evidence-based Medicine (**Supplementary Table 1**).

**Table 1S.** Individual search results for search combinations in Pubmed/Medline

| **Search terms** | **Search Results** |
| --- | --- |
| ((("myocardic"[All Fields] OR "myocarditis"[MeSH Terms]) OR "myocarditis"[All Fields]) OR "myocarditides"[All Fields]) AND "covid"[All Fields] OR "SARS-CoV-2" | **490** |
| ((("myocardic"[All Fields] OR "myocarditis"[MeSH Terms]) OR "myocarditis"[All Fields]) OR "myocarditides"[All Fields]) AND "coronavirus"[All Fields] | **490** |
| ((("acute"[All Fields] OR "acutely"[All Fields]) OR "acutes"[All Fields]) AND ((("cardiacs"[All Fields] OR "heart"[MeSH Terms]) OR "heart"[All Fields]) OR "cardiac"[All Fields]) AND ((((((((((("injurie"[All Fields] OR "injuried"[All Fields]) OR "injuries"[MeSH Subheading]) OR "injuries"[All Fields]) OR "wounds and injuries"[MeSH Terms]) OR ("wounds"[All Fields] AND "injuries"[All Fields])) OR "wounds and injuries"[All Fields]) OR "injurious"[All Fields]) OR "injury s"[All Fields]) OR "injuryed"[All Fields]) OR "injurys"[All Fields]) OR "injury"[All Fields])) AND "covid"[All Fields] | **317** |
| ((("acute"[All Fields] OR "acutely"[All Fields]) OR "acutes"[All Fields]) AND ((("cardiacs"[All Fields] OR "heart"[MeSH Terms]) OR "heart"[All Fields]) OR "cardiac"[All Fields]) AND ((((((((((("injurie"[All Fields] OR "injuried"[All Fields]) OR "injuries"[MeSH Subheading]) OR "injuries"[All Fields]) OR "wounds and injuries"[MeSH Terms]) OR ("wounds"[All Fields] AND "injuries"[All Fields])) OR "wounds and injuries"[All Fields]) OR "injurious"[All Fields]) OR "injury s"[All Fields]) OR "injuryed"[All Fields]) OR "injurys"[All Fields]) OR "injury"[All Fields])) AND "coronavirus"[All Fields] | **283** |
| (((("cardiacs"[All Fields] OR "heart"[MeSH Terms]) OR "heart"[All Fields]) OR "cardiac"[All Fields]) AND ((("damage"[All Fields] OR "damaged"[All Fields]) OR "damages"[All Fields]) OR "damaging"[All Fields])) AND "covid"[All Fields] | **355** |
| (((("myocardially"[All Fields] OR "myocardium"[MeSH Terms]) OR "myocardium"[All Fields]) OR "myocardial"[All Fields]) AND ((("damage"[All Fields] OR "damaged"[All Fields]) OR "damages"[All Fields]) OR "damaging"[All Fields])) AND "covid"[All Fields] | **130** |

1. **Data extraction and synthesis**

From eligible studies, we extracted publication-related characteristics (i.e., first author, year of publication) and patient-related characteristics. In specific, we recorded demographic parameters and the clinical presentation of myocarditis cases along with laboratory findings (focusing on troponin and B-type Natriuretic Peptide -BNP- levels and inflammatory markers), imaging data from chest Computer tomography (CT) or Chest X-Ray (CXR), available information from coronary angiogram (CA) - either CT or invasive- cardiac echocardiography and CMR results. When available, data from endomyocardial biopsy (EMB) or post-mortem autopsies were included. We also reported administered therapy - both myocarditis related and virus-specific treatment- and the final clinical outcome. We used a harvest plot to summarize the evidence about the differential effect of baseline characteristics, imaging findings and selected treatments on the outcome of COVID-19 related myocarditis.**List of full text articles excluded (n=53) from the qualitative synthesis of eligible cases on COVID-19 and myocarditis.**

1 Basso C, Leone O, Rizzo S, et al. Pathological features of COVID-19-associated myocardial injury: a multicentre cardiovascular pathology study. European heart journal 2020.

2 Yang F, Shi S, Zhu J, et al. Analysis of 92 deceased patients with COVID-19. Journal of medical virology 2020.

3 Yang Q, Xie L, Zhang W, et al. Analysis of the clinical characteristics, drug treatments and prognoses of 136 patients with coronavirus disease 2019. Journal of clinical pharmacy and therapeutics 2020.

4 Yu Y, Xu D, Fu S, et al. Patients with COVID-19 in 19 ICUs in Wuhan, China: a cross-sectional study. Critical care (London, England) 2020;24:219.

5 Zheng Y, Xu H, Yang M, et al. Epidemiological characteristics and clinical features of 32 critical and 67 noncritical cases of COVID-19 in Chengdu. Journal of clinical virology : the official publication of the Pan American Society for Clinical Virology 2020;127:104366.

6 Guo T, Fan Y, Chen M, et al. Cardiovascular Implications of Fatal Outcomes of Patients With Coronavirus Disease 2019 (COVID-19). JAMA cardiology 2020.

7 Yang X, Yu Y, Xu J, et al. Clinical course and outcomes of critically ill patients with SARS-CoV-2 pneumonia in Wuhan, China: a single-centered, retrospective, observational study. The Lancet Respiratory medicine 2020;8:475-81.

8 Shi S, Qin M, Cai Y, et al. Characteristics and clinical significance of myocardial injury in patients with severe coronavirus disease 2019. European heart journal 2020.

9 Shi S, Qin M, Shen B, et al. Association of Cardiac Injury With Mortality in Hospitalized Patients With COVID-19 in Wuhan, China. JAMA cardiology 2020.

10 Deng Q, Hu B, Zhang Y, et al. Suspected myocardial injury in patients with COVID-19: Evidence from front-line clinical observation in Wuhan, China. International journal of cardiology 2020.

11 Huang C, Wang Y, Li X, et al. Clinical features of patients infected with 2019 novel coronavirus in Wuhan, China. Lancet (London, England) 2020;395:497-506.

12 Tian S, Xiong Y, Liu H, et al. Pathological study of the 2019 novel coronavirus disease (COVID-19) through postmortem core biopsies. Modern pathology : an official journal of the United States and Canadian Academy of Pathology, Inc 2020:1-8.

13 Misra DP, Agarwal V, Gasparyan AY, et al. Rheumatologists' perspective on coronavirus disease 19 (COVID-19) and potential therapeutic targets. Clinical rheumatology 2020:1-8.

14 Barton LM, Duval EJ, Stroberg E, et al. COVID-19 Autopsies, Oklahoma, USA. American journal of clinical pathology 2020;153:725-33.

15 Yuan WF, Tang X, Zhao XX. An 'asymptomatic' driver with COVID-19: atypical suspected myocarditis by SARS-CoV-2. Cardiovascular diagnosis and therapy 2020;10:242-3.

16 Zeng JH, Liu YX, Yuan J, et al. First case of COVID-19 complicated with fulminant myocarditis: a case report and insights. Infection 2020:1-5.

17 Radbel J, Narayanan N, Bhatt PJ. Use of Tocilizumab for COVID-19-Induced Cytokine Release Syndrome: A Cautionary Case Report. Chest 2020.

18 Chen L, Upadhya G, Guo US, et al. Novel Coronavirus-Induced Right Ventricular Failure and Point of Care Echocardiography: A Case Report. Cardiology 2020:1-6.

19 Chau VQ, Oliveros E, Mahmood K, et al. The Imperfect Cytokine Storm: Severe COVID-19 with ARDS in Patient on Durable LVAD Support. JACC Case reports 2020.

20 Kir D, Mohan C, Sancassani R. HEART BRAKE-An unusual cardiac manifestation of Coronavirus disease 2019 (COVID-19). JACC Case reports 2020.

21 Behrendt D, Ganz P. Endothelial function. From vascular biology to clinical applications. The American journal of cardiology 2002;90:40l-8l.

22 Oberweis ML, Codreanu A, Boehm W, et al. Pediatric Life-Threatening Coronavirus Disease 2019 With Myocarditis. The Pediatric infectious disease journal 2020.

23 Horowitz RI, Freeman PR, Bruzzese J. Efficacy of glutathione therapy in relieving dyspnea associated with COVID-19 pneumonia: A report of 2 cases. Respiratory medicine case reports 2020;30:101063.

24 Tavazzi G, Pellegrini C, Maurelli M, et al. Myocardial localization of coronavirus in COVID-19 cardiogenic shock. European journal of heart failure 2020;22:911-5.

25 Loghin C, Chauhan S, Lawless SM. Pseudo acute myocardial infarction in a young COVID-19 patient. JACC Case reports 2020.

26 Blaivas M. Unexpected finding of myocardial depression in 2 healthy young patients with COVID-19 pneumonia: possible support for COVID-19-related myocarditis. J Am Coll Emerg Physicians Open 2020;1:375-8.

27 Bradley BT, Maioli H, Johnston R, et al. Histopathology and ultrastructural findings of fatal COVID-19 infections in Washington State: a case series. Lancet (London, England) 2020;396:320-32.

28 Cuomo G, Menozzi M, Carli F, et al. Acute myocarditis as the main clinical manifestation of SARS-CoV 2 infection. Infect Dis Rep 2020;12:8609.

29 Derveni V, Kaniaris E, Toumpanakis D, et al. Acute life-threatening cardiac tamponade in a mechanically ventilated patient with COVID-19 pneumonia. IDCases 2020;21:e00898.

30 Ho JS, Sia CH, Chan MY, et al. Coronavirus-induced myocarditis: A meta-summary of cases. Heart Lung 2020;49:681-5.

31 Juusela A, Nazir M, Gimovsky M. Two cases of coronavirus 2019-related cardiomyopathy in pregnancy. Am J Obstet Gynecol MFM 2020;2:100113.

32 Khalid Y, Dasu N, Dasu K. A case of novel coronavirus (COVID-19)-induced viral myocarditis mimicking a Takotsubo cardiomyopathy. HeartRhythm Case Rep 2020;6:473-6.

33 Mansoor A, Chang D, Mitra R. Rhythm, Conduction and ST elevation with COVID-19: Myocarditis or Myocardial Infarction? HeartRhythm Case Rep 2020.

34 Naderi N, Ansari Ramandi MM, Baay M, et al. Cardiovascular patients in COVID-19 era, a case series, an experience from a tertiary cardiovascular center in Tehran, Iran. Clin Case Rep 2020.

35 Murillo F, Ramos G, Del Pozo JL, et al. SARS-CoV-2 Infection with Associated Rhabdomyolysis and Probable Myocarditis. Eur J Case Rep Intern Med 2020;7:001867.

36 Naneishvili T, Khalil A, O'Leary R, et al. Fulminant myocarditis as an early presentation of SARS-CoV-2. BMJ Case Rep 2020;13.

37 Rehman M, Gondal A, Rehman NU. Atypical Manifestation of COVID-19-Induced Myocarditis. Cureus 2020;12:e8685.

38 Sardari A, Tabarsi P, Borhany H, et al. Myocarditis detected after COVID-19 recovery. European heart journal cardiovascular Imaging 2020.

39 Wenzel P, Kopp S, Göbel S, et al. Evidence of SARS-CoV-2 mRNA in endomyocardial biopsies of patients with clinically suspected myocarditis tested negative for COVID-19 in nasopharyngeal swab. Cardiovascular research 2020;116:1661-3.

40 Chiu JS, Lahoud-Rahme M, Schaffer D, et al. Kawasaki Disease Features and Myocarditis in a Patient with COVID-19. Pediatr Cardiol 2020:1-3.

41 Bordet J, Perrier S, Olexa C, et al. Paediatric multisystem inflammatory syndrome associated with COVID-19: filling the gap between myocarditis and Kawasaki? Eur J Pediatr 2020:1-8.

42 Grimaud M, Starck J, Levy M, et al. Acute myocarditis and multisystem inflammatory emerging disease following SARS-CoV-2 infection in critically ill children. Ann Intensive Care 2020;10:69.

43 Dolhnikoff M, Ferreira Ferranti J, de Almeida Monteiro RA, et al. SARS-CoV-2 in cardiac tissue of a child with COVID-19-related multisystem inflammatory syndrome. Lancet Child Adolesc Health 2020;4:790-4.

44 Purohit R, Kanwal A, Pandit A, et al. Acute Myopericarditis with Pericardial Effusion and Cardiac Tamponade in a Patient with COVID-19. Am J Case Rep 2020;21:e925554.

45 Lara D, Young T, Del Toro K, et al. Acute Fulminant Myocarditis in a Pediatric Patient With COVID-19 Infection. Pediatrics 2020;146.

46 El-Assaad I, Hood-Pishchany MI, Kheir J, et al. Complete Heart Block, Severe Ventricular Dysfunction, and Myocardial Inflammation in a Child With COVID-19 Infection. JACC Case reports 2020;2:1351-5.

47 Kesici S, Aykan HH, Orhan D, et al. Fulminant COVID-19-related myocarditis in an infant. European heart journal 2020;41:3021.

48 Amoozgar B, Kaushal V, Mubashar U, et al. Symptomatic pericardial effusion in the setting of asymptomatic COVID-19 infection: A case report. Medicine (Baltimore) 2020;99:e22093.

49 Taza F, Zulty M, Kanwal A, et al. Takotsubo cardiomyopathy triggered by SARS-CoV-2 infection in a critically ill patient. BMJ Case Rep 2020;13.

50 Farina A, Uccello G, Spreafico M, et al. SARS-CoV-2 detection in the pericardial fluid of a patient with cardiac tamponade. Eur J Intern Med 2020;76:100-1.

51 Beri A, Kotak K. Cardiac injury, arrhythmia, and sudden death in a COVID-19 patient. HeartRhythm Case Rep 2020;6:367-9.

52 Seecheran R, Narayansingh R, Giddings S, et al. Atrial Arrhythmias in a Patient Presenting With Coronavirus Disease-2019 (COVID-19) Infection. J Investig Med High Impact Case Rep 2020;8:2324709620925571.

53 Parsova KE, Pay L, Oflu Y, et al. A rare presentation of a patient with COVID-19: Cardiac tamponade. Turk Kardiyol Dern Ars 2020;48:703-6.

54. Garau G, Joachim S, Duliere GL, Melissopoulou M, Boccar S, Fraipont V, Dugauquier C, Troisfontaines P, Hougrand O, Delvenne P, Hoffer E. Sudden cardiogenic shock mimicking fulminant myocarditis in a surviving teenager affected by severe acute respiratory syndrome coronavirus 2 infection. ESC Heart Fail 2020.

55. Ciuca C, Fabi M, Di Luca D, Niro F, Ghizzi C, Donti A, Balducci A, Rocca A, Zarbo C, Gargiulo GD, Lanari M. Myocarditis and coronary aneurysms in a child with acute respiratory syndrome coronavirus 2. ESC Heart Fail 2020.

56. Gay HC, Sinha A, Michel E, Mozer AB, Budd A, Feinstein MJ, Benzuly KH, Al-Qamari A, Pawale AA, Vorovich EE. Fulminant myocarditis in a patient with coronavirus disease 2019 and rapid myocardial recovery following treatment. ESC Heart Fail 2020;**7**(6):4367-70.

57. Tiwary T, Baiswar S, Jinnur P. A Rare Case of COVID-19 Myocarditis With Cardiac Tamponade in a Young Diabetic Adult With Renal Failure. Cureus 2020;**12**(11):e11632.

58. Yokoo P, Fonseca E, Sasdelli Neto R, Ishikawa WY, Silva MMA, Yanata E, Chate RC, Nunes Filho ACB, Bettega M, Fernandes JRC, Tarasoutchi F, Szarf G. COVID-19 myocarditis: a case report. Einstein (Sao Paulo) 2020;**18**:eRC5876.

59. Laurence C, Haini M, Thiruchelvam T, Derrick G, Burch M, Yates RWM, Simmonds J. Endomyocardial Biopsy in a Pediatric Patient With Cardiac Manifestations of COVID-19. Circ Heart Fail 2020;**13**(11):e007384.

60. Kohli U, Meinert E, Chong G, Tesher M, Jani P. Fulminant myocarditis and atrial fibrillation in child with acute COVID-19. J Electrocardiol 2020.

61. Oleszak F, Maryniak A, Botti E, Abrahim C, Salifu MO, Youssef M, Henglein VL, McFarlane SI. Myocarditis Associated With COVID-19. Am J Med Case Rep 2020;**8**(12):498-502.

1. **Unpublished case-report**

In our unpublished experience, we describe herein a peculiar case from Bergamo, Italy. A 43-year-old woman with no significant previous illness presents to the hospital for chest pain and on admis-sion, a 12-lead ECG showed an ST-segment elevation in inferior and lateral leads. The echocardio-gram reveals severe LV dysfunction (left ventricular ejection fraction 25%) Blood tests documented normal white and red cell counts, mild acute kidney and liver injury and mild increased high sen-sitivity C-reactive protein (1.4 mg/dL). International Normalized Ratio (INR) and partial thrombo-plastin time (PTT) were in normal range. High sensitivity troponin I was 11,795 ng/L. Chest radiog-raphy showed pulmonary congestion, without signs of interstitial pneumonia. Nasopharyngeal swab was positive for SARS-CoV2 infection. The clinical course was characterised by an irreversible multi-organ failure. Plasminogen Activator Inhibitor (PAI-1), Prothrombin activation fragments (F1+2), and plasma level of von Willebrand factor were markedely increased. Despite intensive management and hemodynamic support, a rapid progression to cardiogenic shock led to death on the second day after admission.

1. **Supplementary Table 1**

| **Supplementary Table 1.** Quality Rating Scheme for Studies and Other Evidence | |
| --- | --- |
| **Inciardi et al ^3^** | 5 Opinion of respected authorities; case reports |
| **Sala et al ^4^** | 5 Opinion of respected authorities; case reports |
| **Doyen et al ^5^** | 5 Opinion of respected authorities; case reports |
| **Varga et al ^6^** | 5 Opinion of respected authorities; case reports |
| **Luetkens et al ^7^** | 5 Opinion of respected authorities; case reports |
| **Hu et al ^8^** | 5 Opinion of respected authorities; case reports |
| **Paul et al ^9^** | 5 Opinion of respected authorities; case reports |
| **Coyle et al ^10^** | 5 Opinion of respected authorities; case reports |
| **In-Cheol Kim et al ^11^** | 5 Opinion of respected authorities; case reports |
| **Craver et al ^12^** | 5 Opinion of respected authorities; case reports |
| **Trogen et al. ^13^** | 5 Opinion of respected authorities; case reports |
| **Besler et al. ^14^** | 5 Opinion of respected authorities; case reports |
| **Gnecchi et al. ^15^** | 5 Opinion of respected authorities; case reports |
| **Garot et al. ^16^** | 5 Opinion of respected authorities; case reports |
| **Al-Assaf et al. ^17^** | 5 Opinion of respected authorities; case reports |
| **Salamanca et al. ^18^** | 5 Opinion of respected authorities; case reports |
| **Bonnet et al. ^19^** | 5 Opinion of respected authorities; case reports |
| **De Vita et al. ^20^** | 5 Opinion of respected authorities; case reports |
| **Pavon et al. ^21^** | 5 Opinion of respected authorities; case reports |
| **Ford et al. ^22^** | 5 Opinion of respected authorities; case reports |
| **Richard et al. ^23^** | 5 Opinion of respected authorities; case reports |
| **Warchol et al. ^24^** | 5 Opinion of respected authorities; case reports |
| **Jacobs et al. ^25^** | 5 Opinion of respected authorities; case reports |
| **Hua et al. ^26^** | 5 Opinion of respected authorities; case reports |
| **Cizgici et al. ^27^** | 5 Opinion of respected authorities; case reports |
| **Hussain et al. ^28^** | 5 Opinion of respected authorities; case reports |
| **Spano et al. ^29^** | 5 Opinion of respected authorities; case reports |
| **Dalen et al. ^30^** | 5 Opinion of respected authorities; case reports |
| **Labani et al. ^31^** | 5 Opinion of respected authorities; case reports |
| **Khatri et al. ^32^** | 5 Opinion of respected authorities; case reports |
| **Albert et al. ^33^** | 5 Opinion of respected authorities; case reports |
| **Nicol et al. ^34^** | 5 Opinion of respected authorities; case reports |
| **Gauchotte et al. ^35^** | 5 Opinion of respected authorities; case reports |
| **Caraffa et al. ^36^** | 5 Opinion of respected authorities; case reports |
| **Iqbal et al.^37^** | 5 Opinion of respected authorities; case reports |
| **Othenin et al. ^38^** | 5 Opinion of respected authorities; case reports |
| **Escher et al. ^39^** | 5 Opinion of respected authorities; case series |
| According to the Oxford Centre for Evidence-based Medicine's Levels of Evidence and Grades of Recommendation | |

**Supplementary Table 2. Summary of published case reports on Covid-19 and acute myocarditis**

| **CLINICAL CASES** | Inciardi et al,  JAMA Cardiol^3^ | Sala et al,  European Heart  Journal^40^ | Doyen et al,  The Lancet^41^ | Varga et al,  [The](http://www.thelancet.com) Lancet^42^ | Luetkens et al. Circ Cardiovasc  Imaging^43^ |
| --- | --- | --- | --- | --- | --- |
| **CLINICAL PICTURE** | 53F, severe fatigue, BP 90/50mmHg, HR 100bpm, fever and cough 1-week prior admission. | 43F, 3 days of chest pain and dyspnoea. T 37.7, BP120/80mmHg, HR 79bpm, SO2 89%, ronchi lung bases | 69M, 7 days after fever 39°C, vomiting, diarrhoea and dyspnoea, was admitted with ARDS requiring ITU for mechanical ventilation | 71M, BG of renal transplant, CAD, HTN. Required mechanical ventilation, had multi organ failure | 79M, history of asthma, admitted for fatigue, dyspnoea and recurrent syncope. T 35.6 C, HR 75bpm, BP, SO294%, ARDS requiring ITU for mechanical ventilation |
| **ETHNICITY/RACE** | Italian, Caucasian | Italian | Italian, Caucasian | Not available | Not available  (Authors from Germany) |
| **COMORBIDITIES** | No previous history of CVD | Unremarkable | Hypertension under treatment | CAD, HTN and renal transplant recipient | Asthma  No history of CVD |
| **ACUTE BLOODS** | NTproBNP 8,456 pg/ml, Troponin T raise to 590 ng/L mild CRP raise | NTproBNP 512 pg/ml, Troponin T 135ng/L  CRP raise | Troponin I 9,002 ng/L | NTproBNP: 10’456ng/l, Troponin T: 51ng/l  CRP. D-dimers raised | NTproBNP 1,178ng/L, Troponin Τ 63.5 ng/L  Raised CRP |
| **ACUTE ECG** | Mild infero-lateral ST elevation, low voltage limbs leads | Low atrial rhythm, mild ST elevation V1-V2, aVR, diffuse U waves | LVH and diffuse T waves inversion | Not available | Reported as normal |
| **CXR/CT** | Normal | CXR: Bilateral opacities (interstitial lung disease) | CT: bilateral ground-glass opacities and condensations | Not available | CXR: Reported as normal  CT: ground-glass peripheral infiltrates in the left upper lobe, pleural and pericardial effusion |
| **CTCA/Angiogram** | Normal | Normal coronaries, no PE. 3D dynamic reconstruction: EF52%, basal hypokinesis, good apical contraction  (reverse Takotsubo pattern) | Normal | Not available | Not performed |
| **ECHO** | Normal LV size, LVH ( 14mm IVS and PW); EF40%  Repeat echo ( day 6) Normal wall thickness, EF44% | Inferolateral hypokinesia, EF43% | Mild LVH, preserved EF no RWMA | Preserved LVEF,  but a severely enlarged left atrium (59ml/m2) indicating longstanding diastolic  dysfunction. | Reported as normal |
| **CMR** | LVH, diffuse hypokinesis , abnormal STIR and T2 mapping (oedema); LGE in the entire biventricular walls | (day7 from admission) EF 64%, mild hypokinesia basal -mid LV, co-localised diffuse oedema , pseudo-hypertrophy. Oedema on STIR and T2 mapping | Subepicardial LGE of the apex and inferolateral wall | Not available | (day 10 from admission) Normal LV size, EF 49% with global hypokinesia. Pericardial effusion (10mm), diffuse oedema, no LGE but increased T1 relaxation time ( +Lake Luise criteria 2018) |
| **EMB**  **Postmortem** | - | T-lymphocitic inflammation (CD3+>7/mm2), huge interstitial oedema, no necrosis. Microvascular abnormalities. No viral Genoma in the myocytes ( virus negative lymphocytic myocarditis) | - | Postmortem:  accumulation of inflammatory cells associated with endothelium, as well as apoptotic bodies, in the heart | - |
| **DRUG**  **THERAPY** | Dobutamine, kanrenone, furosemide, bisoprolol, HCQ, lopinavir/ritonavir, methylprednisolone | lopinavir/ritonavir. and HCQ | fondaparinux, and dual anti platelet for suspected ACS  Hydrocortisone form day 11 for 9 days | Not available | Medical treatment of heart failure (initiated after myocarditis) |
| **OUTCOME** | Clinical and instrumental stabilization | Discharged with no symptoms and preserved cardiac function | Discharged from ICU after 3months | Death | Recovered |

| **CLINICAL CASES** | Hu et al. European Heart Journal ^8^ | Paul et al. Eur. Heart J. Cardiovasc Imaging ^9^ | Coyle et al JACC Case rep ^10^ | In-Cheol Kim et al.  Eur. Heart J. ^11^ | Craver et al,  Fetal Pedia.Pathol. ^12^ |
| --- | --- | --- | --- | --- | --- |
| CLINICAL PICTURE | 37F, chest pain and dyspnoea for 3days, accompanied by diarrhoea, BP 80/50mmHg | 35M, Hx of obesity, chest pain and fatigue, no other symptoms | 57M, shortness of breath, fever, cough, myalgias, decreased appetite, nausea, and diarrhea for one week. Day 3: ARDS and mechanical ventilation. | 21F, fever, cough , diarrhoea, dyspnoea | 17M, severe headaches, dizziness, nausea and vomiting, for 2 days, Day 3: collapsed and transferred pulseless to the Emergency Department |
| ETHNICITY/RACE | Not available  (Chinese Authors) | Not available  (French Authors) | Not available  (USA Authors) | Not available  (Korean Authors) | African American |
| COMMORBIDITIES | Not available | Obesity | Hypertension under treatment | Not available, probably none | Not available |
| ACUTE BLOODS | NTproBNP 21,025 ng/ml, Troponin T> 10,000 ng/L | Troponin I (peak) 2,885 ng/L | NTproBNP 1,300 pg/ml, Troponin I 7.33 ng/mL | NTproBNP1,929pg/ml  Troponin I 1,260ng/L | Not available |
| ACUTE ECG | ST elevation in III, AVF | Repolarization changes in the precordial ECG leads | sinus tachycardia without ST/T wave changes | Non-specific intraventricular conduction delay and PCVs | In cardiac arrest at presentation |
| CXR/CT | CXR: Significant enlargement of the heart  CT: pulmonary infection, enlarged heart, and pleural effusion | CT: Normal | CXR and CT:  progressive bilateral patchy interstitial opacities | CXR: Multi-focal consolidation and cardiomegaly; CT multifocal consolidation and ground-glass opacification in both lungs in the lower lobe and a peripheral dominant distribution | Not available |
| CTCA/Angiogram | CTCA: No coronary stenosis | Not performed | Not performed | Cardiac CT : normal coronaries, LVH (oedema)and subendocardial perfusion defect on the lateral wall | Not available |
| ECHO | LVEDD: 58mm, LAD: 39mm, RVEDD:25mm,RA: 48mm, LVEF:27%,2mm pericardial effusion | Normal systolic function with no pericardial effusion | Diffuse hypokinesis with relative apical sparing, LVEF:35-40% | Severe LVSD | Not available |
| CMR | Not performed | Late sub-epicardial enhancement predominating in the inferior and lateral walls | Diffuse  bi-ventricular and bi-atrial oedema with a small area of late gadolinium enhancement | High signal intensity on STIR images, LVH, elevated native T1 mapping and ECV in mid-septum and lateral wall. Extensive transmural LGE of the lateral and apical walls | Not available |
| EMB  PostMortem | - | - | - | - | Postmortem: Diffuse inflammatory infiltrates with prominent eosinophils, mainly in the interstitium of both ventricles and associated with multiple foci of myocyte necrosis. Pericardial fluid |
| DRUG  THERAPY | methylprednisolone, norepinephrine, diuretic (toracemide and furosemide), milrinone,piperacillin sulbactam | ramipril 5mg,  bisoprolol 5mg | HCQ, azithromycin, ceftriaxone, methylprednisolone, colchicine, milrinone, tocilizumab, aldose reductase inhibitor (1500 mg every 12 hours for 14 days) | Not available | None |
| OUTCOME | Patient recovered with normal LV function | Patient recovered | Discharged from hospital | Not available | Sudden death |

| **CLINICAL**  **CASES** | Trogen, et al. The Pediatric Infectious Disease Journal ^13^ | Besler et al. American Journal of Emergency Medicine^14^ | Gnecchi et al.Lancet ^15^ | Garot et al. JACC Case Reports ^16^ |
| --- | --- | --- | --- | --- |
| **CLINICAL PICTURE** | 17M, 7 days of fever, gastrointestinal symptoms, neck pain. Admitted in ICU for septic shock and myocarditis | 20M, New onset febrile sensation and chest pain | 16M, intense chest pain-radiating to left arm, 38.3°C fever the day before. | 18M, cough fever (38.5°C),  fatigue and myalgia. Day 3 ARDS, intubation |
| **ETHNICITY**  **/RACE** | (not available , authors from US, New York) | (not available , authors from Turkey) | (not available , authors from Italy) | (not available , authors from France) |
| **COMORB-IDITIES** | Obesity  (BMI>30) , mild asthma , spondylolysis | No previous history of CVD is mentioned | No medical history | No medical history |
| **ACUTE BLOODS** | NTproBNP: 2,124pg/ml, TnI: 6.17 ng/L CRP raise | NTproBNP: 127ng/L, TnI 0.572 ng /mL, CK-MB 9.08 μg/L, CRP 0.0812 g/L | TnI: 9,449 ng/L, CRP: 32.5 mg/L | NT-proBNP: 11,719  pg/mL, troponin 11,716 UI/mL, CRP 351 mg/L |
| **ACUTE ECG** | Sinus tachycardia , TWI inferiorly | Not available | Inferolateral ST-segment elevation | Sinus tachycardia, TWI V2-V4 |
| **CXR/CT** | Hazy ground glass opacities at the lower lobes bilaterally with no focal consolidation.  Mild LVSD | Focal consolidation on the upper zone of left lung / sub-pleural consolidation containing air bronchogram with a halo of ground-glass opacification in the left upper lobe | Normal | Diffuse peripheral opacity “crazy paving” |
| **CTCA/**  **Angiogram** | Not performed | Not available | Not available | Not available |
| **ECHO** | Mild LVSD | Not available | Hypokinesia of the inferior and inferolateral segments of the LV, with a preserved EF (52%) | Diffuse hypokinesis EF (30%), LV enlarged (32 mm/m² EDD) and increased wall thickness (IVS and PW 14 mm) |
| **CMR** | Normal LV size, LVEF (40%). Normal RV size, RVEF of 39%. Mid-wall LGE at the inferior LV–RV junction corresponding to an area of increased T2 signal, as well as an area of hypokinesia. | Normal LV function, volumes, and mass (EF 64%. T2 STIR sequence revealed a subepicardial high signal intensity in the mid posterolateral wall of the LV. subepicardial LGE of the posterolateral wall in the mid ventricle at 5 and 10 min after contrast | T2-weighted STIR image shows a  subepicardial band-like high signal indicating patchy oedema involving the  whole of the lateral wall. LGE image shows a high intensity signal | Increased LV  wall thickness (14 mm), volumes, marked diffuse hypokinesis (LVEF 33%. STIR images indicated marked extensive hypersignal of the LV basal posterolateral wall. LGE images demonstrated nodular subepicardial enhancement of the LV basal posterolateral wall |
| **EMB**  **Postmortem** | Not performed | Not available | Not available | Not available |
| **DRUG**  **THERAPY** | HCQ (interrupted for prolonged QT) piperacillin/tazobactam (stopped for normal blood future after 48h) | HCQ, azithromycin, ceftriaxone,  tigecycline, favipiravir, and colchicine | Aspirin, ibuprofen, HCQ and antiviral therapy | Paracetamol, HCQ, noradrenalin, cefotaxime and rovamycine |
| **OUTCOME** | Recovered and discharged | Recovered and discharged | Recovered and discharged | Recovered and discharged |

| **CLINICAL CASES** | Al-Assaf et al. HeartRhythm Case Rep^17^ | Salamanca et al JACC Cardiovasc Imaging^18^ | Bonnet et al. JACC Heart Fail ^19^ | De Vita et al. Echocardiography ^20^ |
| --- | --- | --- | --- | --- |
| **CLINICAL PICTURE** | 58M, isolation due to close contact with a case of COVID-19. On day 9, asymptomatic bradycardia (40 beats per minute). | 44M, severe bradycardia, hypotension, and signs of peripheral hypoperfusion. Previously presented at the ED with fever, dry cough, diarrhea, and myalgia. | 27M, admitted to the hospital due to respiratory distress. | 35F, worsening fatigue, dyspnea on minimal exertion, and orthopnea in the previous week without fever |
| **ETHNICITY/RACE** | (not available , authors from the United Arab Emirates) | (not available, authors from Spain) | (not available, authors from France) | (not available, Authors from Italy) |
| **COMORBIDITIES** | HTN | Not available | No previous medical history | No previous medical Hx. Chilbirth 1 month ago |
| **ACUTE BLOODS** | Normal ranges of inflammatory markers and cardiac biomarkers. | NTproBNP: 24,167 pg/ml, TnT 745 ng/l, CK-MB 30 U/l | NTproBNP: 9,300 pg/ml, TnI: 100 ng/l | NTproBNP: 6608 ng/L, TnT 37 ng/l |
| **ACUTE ECG** | Sinus bradycardia, no ST-T changes | 3^rd^ degree atrioventricular block | Not available | Diffuse ST changes with negative T-waves in leads V3-V6 |
| **CXR/CT** | Normal | Signs of bilateral pneumonia | Not available | Pleural effusion and pulmonary congestion/ Interstitial and alveolar thickening in the right middle and both inferior lobes, bilateral pleural and pericardial effusion, cardiomegaly, and subsegmental pulmonary embolism |
| **CTCA/**  **Angiogram** | 70% disease in the proximal LAD artery, no other significant lesion | Normal coronary arteries | Not available | Not available |
| **ECHO** | Unremarkable study showing only a mildly dilated ascending aorta. | Non-dilated but globally and severely dysfunctional LV (LVEF 15%) | Enlarged LV with impaired LVEF of 20% | Dilated LV, diffuse marked hypokinesis of LV walls with severe systolic and diastolic dysfunction (biplane LVEF= 20%), moderate mitral regurgitation, mild RV dilation and dysfunction, and pericardial effusion (10 mm). LV apical thrombus |
| **CMR** | T1 mapping showing a high value of 1062.  T2 mapping showing an abnormal value of 57 | Non-dilated LV with normal systolic function (LVEF 75%). Native T1 (mean, 1,120 ms), T2 signal intensity ratio, and extracellular volume (mean, 36%) were diffusely increased with slightly less involvement of the inferolateral wall. LGE was negative | Isolated ventricular non-compaction | Oval-shaped, enlarged LV and RV, with diffuse hypokinesis and reduced systolic function (LVEF = 17%, RVEF= 19%).  LV apical thrombus. Pericardial and pleural effusion. |
| **EMB**  **Postmortem** | Not available | EMB without necrosis inflammation, or fibrosis (HEx200). Isolated intersticial infiltrate with lymphocytes CD3+ | Not available | Not available |
| **DRUG**  **THERAPY** | Enoxaparin, amlodipine | Methylprednisolone, tocilizumab, HCQ, azithromycin, and lopinavirritonavir | Diuretics | Low-molecular weight heparin, furosemide, spironolactone, bisoprolol, ramipril, ethacrynic acid |
| **OUTCOME** | Scheduled a permanent pacemaker implant. Discharged in stable condition. | Recovery and discharged | Recovery and discharged | Recovered and discharged. |

| **CLINICAL CASES** | Pavon et al. August 2020 Can J Cardiol ^21^ | Ford et al. August 2020 Clin Pract Cases Emerg Med ^22^ | Richard et al. July 2020 Cureus ^23^ | Warchol et al. may 2020 Pol Arch Intern Med ^24^ | Jacobs et al. September 2020 ESC Heart Fail ^25^ |
| --- | --- | --- | --- | --- | --- |
| **CLINICAL PICTURE** | 64M, presented with fever and cough | 53M, six-day history of malaise  and fever | 28F, lethargic and covered in coffee ground emesis at home | 74M, presented with new-onset VT(electrical cardioversion). Subsequently, symptomatic bradycardia and hypotension were observed. No fever or any symptoms of respiratory infection | 48M, 7‐day history of fever, diarrhea cough, dysosmia, and dyspnea. Intubation due to hypoexemia. |
| **ETHNICITY/RACE** | (not available, Authors from Switzerland) | (not available Authors from Sacramento, USA) | Caucasian | (not available, authors from Poland) | (not available, Authors from Belgium) |
| **COMORBIDITIES** | Isolated pulmonary  sarcoidosis and epilepsy | Dyslipidemia | DM type 1,  diabetic gastroparesis, asthma, anxiety, depression with multiple previous episodes of diabetic ketoacidosis and recent COVID-19 infection | Atrial fibrillation, catheter ablation performed 3 times, HTN, type 2 diabetes, and hypothyroidism | HTN |
| **ACUTE BLOODS** | TnT: 263 ng/L, CRP: 466 mg/L | BNP 588 pg/mL, TnT normal | troponin 46 ng/mL, CRP 2.47 mg/dL | NTproBNP: 2451 ng/L, TnT 72 ng/L, CRP: 94 mg/L, | NTproBNP, 9,223 pg/mL, TnI 14,932 ng/L |
| **ACUTE ECG** | Normal | Wide-complex, irregular tachycardia with a LBBB morphology, , as well as a long QT interval | Wide complex  tachycardia and accelerated junctional rhythm. ST depression in the latera leads and ST elevation in leads I and aVL | Ventricular tachycardia | QRS widening and a positive  deflection at the end of the T wave |
| **CXR/CT** | Bilateral reticulation and ill-defined opacities,/ minimal ground glass opacities in th right lung | Left lower lobe consolidation | Suspicious for  COVID-19 | Not available | Multiple patchy ground‐glass opacifications in all lung fields |
| **Angiogram/ CTCA** | Exclusion of significant epicardial  coronary stenoses | Not available | Normal left and right coronary arteries, elevated filling pressures and reduced cardiac index (1.9) | Not available | Not available |
| **ECHO** | LVEF 47% | Mild LV dilation with hypokinesis (EF 15%). New transthoracic echo revealed LV thrombus and worsening LV dilation. | LVEF 26-30% and mild mitral  regurgitation | Not available | Hyperdynamic ventricular function (inotropes). IVS 12mm, PW 11mm, LV EDD 48mm |
| **CMR** | Reduced LV systolic function (LVEF: 42%) with mild hypokinesia of the  lateral wall. T2-mapping sequences showed myocardial oedema  .Subepicardial LGE in the anterior IVS and in the inferior and inferolateral walls | LV dilation with global hypokinesis,  increased T2 signal, hyperemia, and edema | Sub-epicardial  myocardial fibrosis and necrosis in the basal to mid anteroseptal  and anterior walls. There is hyperemia  and myocardial oedema in the mid-anteroseptum | LA enlargement and global LV hypokinesia with EF of 20%. LGE: patchy, and linear non-ischemic pattern of fibrosis localised sub-epicardially and intramurally in the basal and mid‑cavity segments of the inferior and inferolateral wall and in the apical segments of the inferior wall | Not available |
| **EMB**  **Postmortem** | Not available | Not available | Not available | Not available | Post Mortem:Hypertrophic  cardiac tissue with patchy muscular, sometimes perivascular,  and slightly diffuse interstitial mononuclear inflammatory infiltrates,  dominated by lymphocytes. Positive immunohistochemical  staining with E06 in morphologically  degenerating and necrotic cardiomyocytes adjacent to the  infiltrate of lymphocytes. |
| **DRUG**  **THERAPY** | Piperacillin/tazobactam | Amiodarone load, ceftriaxone/azithromycin, tissue plasminogen activator, warfarin | Vancomycin and piperacillin-tazobactam, dobutamine, heparin and methylprednisone | Metoprolol, magnesium infusion, amiodarone, azithromycin, oseltamivir | HCQ, azithromycin, noradrenaline, adrenaline, and dobutamine |
| **OUTCOME** | Recovered and discharged. | Recovered and discharged. | Recovered and discharged. | Transferred to a COVID-19 special hospital, in good general condition. | Patient passes away due to refractory shock |

| **CLINICAL**  **CASES** | Hua et al. June 2020 Eur Heart J ^26^ | Cizgici et al. July 2020 Am J Emerg Med ^27^ | Hussain et al. July 2020 Cureus ^28^ | Spano et al. July 2020 Int J Cardiovasc Imaging ^29^ |
| --- | --- | --- | --- | --- |
| **CLINICAL PICTURE** | 47F, Chest pain, breathlessness, dry cough, and subjective fevers | 78M, chest pain and shortness of breath. Developed ARDS. Intubation | 51M, dry cough, fatigue, dyspnea, and a fever along with multiple episodes of epigastric pain and nausea | 49M, new-onset of dyspnea NYHA 3, general weakness, intermittent epigastric pain and nocturia without orthopnea nor fever. COVID-19 a month before. Positive IgG |
| **ETHNICITY/RACE** | Afro-Caribbean | ( Not available, Authors from Turkey) | Italian | ( Not available, Authors from Switzerland) |
| **COMORB-**  **IDITIES** | No risk factors for CVD | Not available | HTN | No risk factors for CVD |
| **ACUTE**  **BLOODS** | TnT: 225 ng/l | TnI: 998ng/l, CRp: 94.6mg/L | Normal troponin 0.29 ng/ml, CK-MB 20.1 ng/ml, NTproBNP: 1287 pg/ml | elevated CRP, troponin and NTproBNP |
| **ACUTE ECG** | Sinus tachycardia, concave inferolateral ST elevation | Atrial fibrillation and concave ST elevation (except aVR) | Normal  2^nd^ : Diffuse ST elevation (I, II, aVL, V1-V6) | T wave inversion V4-V6 |
| **CXR/CT** | Mild pulmonary congestion | Ground glass opacification with consolidation (especially right lower lobe). Bilateral lower lob sub-segmental atelectasis and mild pericardial effusion | Bilateral and peripheral ground-glass and consolidative pulmonary opacities | Left heart congestion |
| **Angiogram/ CTCA** | No obstruction in angiogram in 2017 | Normal coronary arteries | Normal coronary arteris | Not available |
| **ECHO** | Normal LV function and global pericardial effusion with maximum depth of 1.1 cm. 2^nd^ ECHO: maximal depth 2 cm, with evidence of cardiac tamponade | Not available | Enlarged heart with a marked decrease in ventricular systolic  function and an EF 20% | Diffuse hypokinesia with severely depressed left- and right-ventricular function |
| **Pericardiocentisis** | 540mL of serosanguinous fluid and tested negative for COVID-19 | Not available | Not available | Not available |
| **CMR** | Not available | Not available | Not available | Diffuse myocardium and pericardium thickening due to oedema (confirmed with T2 weighted imaging and mapping). Pericardial effusion and tissue characterization revealed diffuse LGE, elevated T1 mapping values and an elevated extracellular volume. Global myocardial strain of all heart chambers was diffusely impaired |
| **EMB/Postmortem** | Not available | Not available | Not available | Not available |
| **DRUG THERAPY** | Not available | Furosemide, beta-blocker and ACEi | Ceftriaxone, vancomycin, remdesivir, HCQ, azithromycin, dobutamine, indomethacin, methylprednisone and colchicine | Not available |
| **OUTCOME** | Not available | Transferred,not available | Deteriorated, no outcome available | Not available |

| **CLINICAL CASES** | Dalen et al. BMJ Case Rep ^30^ | Labani et al. CJC Open ^31^ | Khatri et al. Heart & lung ^32^ | Iqbal et al. Cureus ^37^ |
| --- | --- | --- | --- | --- |
| **CLINICAL PICTURE** | 55F, with near-syncope  and increased body and  chest discomfort for 5 days | 71F, 2-week history of flu-like symptoms, mild fever (38C), chest pain, and a mild decrease of blood oxygen saturation (91%) | 50M, fevers, chills, generalised malaise, non-productive cough, dyspnea for 3-4 days and an episode of near-syncope on the day of presentation. Intubated for ARDS. | 40M, orthopnea, dyspnea, cough |
| **ETHNICITY/RACE** | (not available, Authors from Norway) | ( Not available, Authors from France) | (not available, Authors from NY, USA) | (not available, Authors from USA) |
| **COMORBIDITIES** | No previous medical Hx | Breast cancer treated with surgery, chemotherapy, radiotherapy, and hormonotherapy | HTN, Ischemic stroke | Type 2 Diabetes mellitus |
| **ACUTE BLOODS** | TnT 108 ng/L, NTproBNP  1025 ng/L and CRP 11 mg/ml | TnT: 60 ng/L, BNP: 474 ng/L , CRP, 9 mg/L | Troponin 544ng/L, CK-MB 54.3ng/mL, CRP 11.85mg/dL | TnI 7.3ng/mL, BNP 1710pg/mL |
| **ACUTE ECG** | Sinus tachycardia, insignificant ST-elevation in inferior leads, T wave inversion V1-V3 and low-voltage ECG | Diffuse inverted T waves and elongated QT | sinus tachycardia, ST-elevation in leads II, III, aVF and ST-depression in I, aVL | Sinus tachycardia |
| **CXR/CT** | Normal/ Not performed | Mild bilateral peripheral lower pulmonary lobe ground-glass opacities | Diffuse bilateral patchy opacities | Not available |
| **Angiogram/ CTCA** | Not available | Not available | Right dominant circulation with normal coronary vessels. | No evidence of coronary occlusion |
| **ECHO** | Concentric LV hypertrophy, reduced  LVEF, dilated ICV and pericardial effusion at a maximum of 18 mm, a small RV. Striking hyperechogenic pattern of the endocardium and epicardium. | Infero-septal and infero-apical LV wall hypokinesia, LVEF 56% and a moderate pericardial effusion | Severe global LV systolic dysfunction, RV enlargement, RV systolic dysfunction. A moderate-to-large pericardial effusion was noted anterior to the RV with organizing material | LVEF 21%-25% with moderate pulmonary  hypertension with moderate mitral and tricuspid regurgitation |
| **Pericardiocentisis** | 400 mL of serosal pericardial fluid | Not available | 600cc of serosanguinous fluid was drained. Pericardial fluid studies revealed 31% lymphocytes, 48% monocytes | Not available |
| **CMR** | Myocardial oedema ( T mapping) , increased pericardial thickness and apical and anterolateral myocardial enhancement  consistent with acute perimyocarditis | LV wall motion, normal LVEF 61% and persistence of a mild pericardial effusion. STIR and T2 map showed suggestive of myocardial edema in the basal inferior LV wall. LGE: multiple areas of inferior subepicardial and mid-wall | Not available | Features of myocarditis (inflammatory hyperemia, edema, necrosis,  contractile dysfunction, and accompanying pericardial effusion |
| **EMB/Postmortem** | Not available | Not available | Not available | Not available |
| **DRUG THERAPY** | Dobutamine, and norepinephrine | Not available | Dobutamine, vasopressin, norepinephrine, HCQ, azithromycin, cefepime, vancomycin, methylprednisolone, epinephrine, methylene blue | dexamethasone, remdesevir, lasix, low doses of lisinopril, and metoprolol. |
| **OUTCOME** | Recovered and discharged | Recovered and discharged | Succumbed to multi-organ failure on hospital day four | Recovery and discharge |
| Μ: male, F: female, COVID-19: novel coronavirus 2019 ICU: intensive care unit, ARDS: acute respiratory distress syndrome, BMI: body-mass index, CVD: cardiovascular disease, NTproBNP: N-terminal prohormone of brain natriuretic peptide, TnI: troponin I, TnT: troponin T, CRP: C-reactive protein, CK-MB: creatine kinase myocardial band, TWI: T wave inversion, ECG: electrocardiogram, CXR: chest X-ray, CT: computed tomography, LV: left ventricle, EF: ejection fraction, LVSD: LV systolic dysfunction, RV: right ventricle IVS: intraventricular septum, PW: posterior wall, STIR: short tau inversion recovery LGE: late gadolinium enhancement, EMB: endomyocardial biopsy, HCQ: hydroxychloroquine, HTN: hypertension, LAD: left anterior descending, LCx: left circumflex. EDV: end-diastolic volume, BSA: body surface area, VT: ventricular tachycardia, DM: diabetes mellitus, ACEi: angiotensin converting enzyme inhibitor | | | | |

| **CLINICAL CASES** | **Albert et al.**  **Circulation ^33^** | **Gauchotte et al.**  **Int J Legal Med ^35^** | **Nicol et al. ESC Heart Failure^34^** | **Caraffa et al ESC Heart Failure ^36^** | **Othenin et al. Swiss Medical Weekly ^38^** |
| --- | --- | --- | --- | --- | --- |
| **CLINICAL PICTURE** | 49, M, Fever, myalgias and dyspnea | 69, M, fever, fatigue, abdominal pain | 40, M, odynophagia, left neck pain, fever | 45F,dyspnea,palpilations | 22M, asthenia,  chills, diffuse myalgia, abdominal pain and diarrhoea |
| **ETHNICITY/RACE** | African-American | (Not available, Authors from France) | (Not available, Authors from France) | (Not available, Authors from Italy) | East Africa |
| **COMORBIDITIES** | Smoking | Diabetes mellitus, hypertension, coronary heart disease | Obesity | CKD | Not available |
| **ACUTE**  **BLOODS** | Elevated troponin,  NT-proBNP | Troponin I 8066 pg/mL and CK–MB 2103 UI/L) | Troponin I 485ng/L, BNP 2690 ng/L | TnI 83.000 ng/L, | hsTnI 2718 ng/l |
| **ACUTE ECG** | Sinus tachycardia, no ST-T changes | Normal, no ischemia | Sinus tachycardia | Diffuse ST elevation with tombstone morphology | No signs of acute ischemia |
| **CXR/CT** | No pathological features | Normal | Moderate pleural effusion | N/A | segmental pulmonary embolism with normal lung parenchyma |
| **Angiogram/ CTCA** | N/A | No significant lesions, 2 permeable stents | Normal | Normal | Not available |
| **ECHO** | Globally depressed LVEFof 20% with LVEDD of 5.8 cm, increased wall thickness | Severe and diffuse LV hypokinesia, LVEF=30% | LVEF=45%, cardiac output =3 L/min, subtle hypertrophy and akinesia of posterolateral LV wall, small pericardial effusion opposite. | Severe LV dysfunction | Biventricular dysfunction |
| **Pericardiocentisis** | N/A | N/A | N/A | N/A | Not available |
| **CMR** | N/A | N/A | Mild diastolic dysfunction, with global hypokinesia, T2 indicative of inflammation and LGE suggestive of myocarditis | **N/A** | Not available |
| **EMB/Postmortem** | Inflammatory infiltrates with visualization of viral particles | Post mortem: Multifocal inflammatory infiltration, in both ventricles and septum, composed in its majority of macrophages and lymphocytes. The myocardium was edematous, containing dystrophic cardiomyocytes, without necrosis. Strong presence of anti-SARS-CoV nucleocapsid  protein antibody in the myocardium | Interstitial oedema, small foci of necrosis, interstitial and perivascular infiltrates composed of CD138+, CD79a+ CD20 plasmocytes, CD3+ CD8+ T‐lymphocytes (7cells/mm2), few neutrophils, and a dense and diffuse infiltration by CD163+  macrophages. Positive IgG for SARS-Cov2 | virus‐negative lymphocytic myocarditis | myocardial inflammation with  several foci of myocyte necrosis |
| **DRUG THERAPY** | Tocilizumab, Methyprednizone, IV immunoglobulin, Inotropes, ECMO | Vasopressors, Inotropic support, ECMO, intubation | ACEi,  b-blockers, antibiotics,  anticoagulant and antiarrythmic | Amiodarone, esmolol, lidocaine, methylprednisone, Azathioprine, ECMO | Tocilizumab, IVIG |
| **OUTCOME** | Discharged | Deceased at 6^th^ day of hospitalization | Discharged | Discharge | Recovery and discharge |

| **CLINICAL CASES** | **Escher et al. ESC Heart Failure (patient 1 and 5) ^39^** | |
| --- | --- | --- |
| **CLINICAL PICTURE** | 48M, Sudden onset of high-grade fever and dyspnoea within a  few days | 39M, history of upper airway infection with  headache and fever up to 4 weeks before admission |
| **ETHNICITY/RACE** | German | German |
| **COMORBIDITIES** | N/A | N/A |
| **ACUTE**  **BLOODS** | Hs-Troponin (pg/mL) 3264, BNP (pg/mL) 12 232 | Hs-Troponin (pg/mL) 379, BNP (pg/mL) 109 |
| **ACUTE ECG** | N/A | T wave inversion |
| **CXR/CT** | N/A | N/A |
| **Angiogram/ CTCA** | Coronary artery disease excluded | Coronary artery disease excluded |
| **ECHO** | LVEF= 22% | LVEF=55% |
| **Pericardiocentisis** | N/A | N/A |
| **CMR** | **N/A** | Compatible with myocarditis |
| **EMB/Postmortem** | Active myocarditis with CD3+ 106 cell/mm2 | Bordeline myocarditis with CD3+ 18.74 cell/mm2 |
| **DRUG THERAPY** | Cyclophosmamide and steroids, ICU | ICU, heart failure treatment |
| **OUTCOME** | Recovery | Recovery |

**SuSupplementary Table 3.** Ongoing clinical trials on Covid-19 implementing drugs of cardiovascular interest

| **Drugs tested** | **Trial Design** | **Trial Title** | **Locations** | **Endpoint** | **Clinical Gov Number** |
| --- | --- | --- | --- | --- | --- |
| Cardiovascular drugs | | | | | |
| Aspirin, Losartan, Simvastatin | Multinational, open-label, factorial, randomised trial | Coronavirus Response - Active Support for Hospitalised Covid-19 Patients | Nigeria Pakistan | Cause of death | NCT04343001 |
| Ruxolitinib plus simvastatin | Randomized Phase 2 Clinical Trial | Study of Ruxolitinib Plus Simvastatin in the Prevention and Treatment of Respiratory Failure of COVID-19. | N/A | Percentage of patients who develop severe respiratory failure. | NCT04348695 |
| Aspirin 100mg | Phase 2,3 Randomized, Open label, Controlled Trial | Protective Effect of Aspirin on COVID-19 Patients | China | 1. clinical recovery time  2. the time of SARS-CoV2 overcasting | NCT04365309 |
| Aspirin 75mg Clopidogrel 75mg Rivaroxaban 2.5 MG Atorvastatin 40mg Omeprazole 20mg | Prospective Multicentre Randomised Controlled Trial | Preventing Cardiac Complication of COVID-19 Disease With Early Acute Coronary Syndrome Therapy: A Randomised Controlled Trial. | United Kingdom | All-cause mortality at 30 days after admission | NCT04333407 |
| Discontinuation of RAS blocker therapy continuation of RAS blocker therapy | Phase 3, Open label, Parallel design | ACE Inhibitors or ARBs Discontinuation for Clinical Outcome Risk Reduction in Patients Hospitalized for the Endemic Severe Acute Respiratory Syndrome Coronavirus (SARS-CoV-2) Infection: the Randomized ACORES-2 Study | France | Time to clinical improvement from day 0 to day 28 | NCT04329195 |
| Suspension or Maintenance of Angiotensin Receptor Blockers and Angiotensin-converting Enzyme Inhibitors | Phase 3, Open label, Parallel design | Angiotensin Receptor Blockers and Angiotensin-converting Enzyme Inhibitors and Adverse Outcomes in Patients With COVID19 | Brazil | Median days alive and out of the hospital | NCT04364893 |
| Sildenafil | Single Group Assignment | A Pilot Study of Sildenafil in the Treatment of COVID-19 | China | 1. Rate of disease remission 2. Rate of entering the critical stage  3. Time of entering the critical stage | NCT04304313 |
| Anticoagulant drugs | | | | | |
| Enoxaparin Prophylactic/Intermediate Dose Enoxaparin | Phase 2, open-label multi-center randomized active control trial | Full Dose Heparin Vs. Prophylactic Or Intermediate Dose Heparin in High Risk COVID-19 Patients | USA | Composite outcome of arterial thromboembolic events, venous thromboembolic events and all-cause mortality at Day 30 ± 2 days | NCT04401293 |
| Rivaroxaban 20mg/d followed by enoxaparin/unfractionated heparin when needed control group with enoxaparin 40mg/d | Phase 4, Randomized, parallel assigment, | Full Anticoagulation Versus Prophylaxis in COVID-19: COALIZAO ACTION Trial | N/A | mortality, number of days alive, number of days in the hospital and number of days with oxygen therapy at the end of 30 days. | NCT04394377 |
| Enoxaparin Prophylactic Dose Heparin Infusion Heparin SC Enoxaparin/Lovenox Intermediate Dose | Cluster Based Randomized Selection Trial | Intermediate or Prophylactic-Dose Anticoagulation for Venous or Arterial Thromboembolism in Severe COVID-19 (IMPROVE COVID) | USA | Total Number of Patients with Clinically Relevant Venous or Arterial Thrombotic Events in ICU | NCT04367831 |
| Alteplase 50 MG  Alteplase 100 MG | Phase 2a clinical trial, open label, with a modified stepped-wedge design | Fibrinolytic Therapy to Treat ARDS in the Setting of COVID-19 Infection | United States | PaO2/FiO2 improvement from pre-to-post intervention | NCT04357730 |
| Immunosuppressive drugs | | | | | |
| Cyclosporine | Open, Controlled, Randomized Clinical Trial | Clinical Trial to Assess Efficacy of cYclosporine Plus Standard of Care in Hospitalized Patients With COVID19 | Spain | reducing the severity of COVID19 infection in hospitalized patients | NCT04392531 |
| Colchicine | Randomized, Open Label, Clinical Trial | Treatment With COLchicine of Patients Affected by COVID-19: a Pilot Study | Italy | 1. Respiratory failure (mechanical ventilation); 2. other organ failure warranting ICU monitoring and treatment 3. Death | NCT04375202 |
| Colchicine | Randomized, Parallel assigment, open label Clinical Trial | Colchicine to Counteract Inflammatory Response in COVID-19 Pneumonia | Italy | 1. Clinical improvement 2. Hospital discharge | NCT04322565 |
| Colchicine | Double-blind placebo-controlled clinical trial | Colchicine Twice Daily During 10 Days as an Option for the Treatment of Symptoms Induced by Inflammation in Patients With Mild and Severe Coronavirus Disease | USA,  Canada, Spain | 1. Number of patients with improvement in body temperature, myalgia, arthralgia, total lymphocyte count, D-dimer, fibrinogen and ferritin levels 2. Progression to severe disease | NCT04367168 |
| Colchicine | Phase 3, Randomized, controled parallel assigment clinical trial | Colchicine Coronavirus SARS-CoV2 Trial (COLCORONA) | USA, Spain | Number of participants who die or require hospitalization due to COVID-19 infection | NCT04322682 |
| Colchicine | Phase 2, Randomized, Parallel assigment, open label | COlchicine in Moderate-severe Hospitalized Patients Before ARDS to Treat COVID-19 (COMBATCOVID19) | USA | Percentage of Patients requiring supplemental oxygen beyond 8L nasal cannula | NCT04363437 |
| Colchicine | Phase 2, randomized, open label, | The GReek Study in the Effects of Colchicine in Covid-19 cOmplications Prevention (GRECCO-19) | Greece | 1. Clinical deterioration in the semiquantitative ordinal scale suggested by the WHO R&D committee 2. Maximal concentration of cardiac troponin | NCT04326790 |
| Anakinra | Randomized, Parallel assigment, Double-blinded Clinical trial | Early Identification and Treatment of Cytokine Storm Syndrome in Covid-19 | USA | No increase in oxygen requirement and no increase in respiratory support measures | NCT04362111 |
| Anakinra | Randomized, Factorial Assignment, open label REMAP-COVID is a sub-platform of REMAP-CAP that evaluates treatments specific to COVID-19 | Randomized, Embedded, Multifactorial Adaptive Platform Trial for Community- Acquired Pneumonia (REMAP-CAP) | 89 location studies | 1. All-cause mortality 2. Days alive and outside of ICU | NCT02735707 |
| Anakinra | Phase 2, Randomized,Parallel Assignment | CORIMUNO-ANA: Trial Evaluating Efficacy Of Anakinra In Patients With Covid-19 Infection | France | 1. Survival without needs of ventilator utilization at day 14,  2. WHO progression scale ≤ 5, 3. Cumulative incidence of successful tracheal extubation or withdrawal of NIV or high flow, at day 14, 4. Decrease of at least one point in WHO progression scale score | NCT04341584 |
| Anakinra and trimethoprim/sulfamethoxazole | Phase 2, Single Group Assignment | suPAR-guided Anakinra Treatment for Validation of the Risk and Management of Respiratory Failure by COVID-19 (SAVE) | 13 Centers in Greece | The ratio of patients who will not develop serious respiratory failure | NCT04357366 |
| Anakinra OR Tocilizumab | Non-Randomized, Factorial Assignment, Open label, Clinical trial | Personalised Immunotherapy for SARS-CoV-2 (COVID-19) Associated With Organ Dysfunction | Greece (16 centres) | 1. Change of baseline SOFA score 2. Improvement of lung involvement measurements  3. Increase of pO2/FiO2 ratio | NCT04339712 |
| Emapalumab OR Anakinra | Randomized, Parallel assigment, open label Clinical Trial | Efficacy and Safety of Emapalumab and Anakinra in Reducing Hyperinflammation and Respiratory Distress in Patients With COVID-19 Infection. | Italy | proportion of patients not requiring invasive mechanical ventilation or Extracorporeal membrane oxygenation | NCT04324021 |
| Canakinumab | Phase 2, single center, blinded randomized-controlled study | Canakinumab to Reduce Deterioration of Cardiac and Respiratory Function Due to COVID-19 | USA | Time to clinical improvement up to day 14 | NCT04365153 |
| Canakinumab | Observational Study | Observational Study, Use of Canakinumab Administered Subcutaneously in the Treatment COVID-19 Pneumonia | N/A | intensive care treatment | NCT04348448 |
| Canakinumab | Randomized, Double blinded, parallel, | Study of Efficacy and Safety of Canakinumab Treatment for CRS in Participants With COVID-19-induced Pneumonia (CAN-COVID) | Novartis Pharmaceuticals USA, UK, Spain, Germany | Number of patients with clinical response | NCT04362813 |
| Camostat Mesilate | Randomized, Parallel Assignment | The Impact of Camostat Mesilate on COVID-19 Infection | 9 centers in Denmark | Days to clinical improvement from study enrolment | NCT04321096 |
| Camostat  Mesilate  Placebo  Hydroxychloroquine | Randomized, Parallel Assignment | Combination Therapy With Camostat Mesilate + Hydroxychloroquine for COVID-19 | N/A | Not hospitalized,  Time to improvement of 2 categories from admission on a 7-point ordinal scale,  Proportion of participants in each group with normalization of fever  (and 8 more...) | NCT04338906 |
| Tocilizumab | Single Group Assignment | Tocilizumab in COVID-19 Pneumonia (TOCIVID-19) | Italy, 27 centers | One-month mortality, Il-6 levels, lymphocyte number and 7 more | NCT04317092 |
| Tocilizumab | Single Group Assignmen | Clinical Trial to Evaluate the Effectiveness and Safety of Tocilizumab for Treating Patients With COVID-19 Pneumonia | Spain, 47 centers | To calulate the time of intubation  To calculate the time with oxygen therapy  To calculate the time with Non-invasive mechanical ventilation, 13 more | NCT04445272 |
| Drug: Remdesivir  Drug: Tocilizumab  Drug: Placebo | Randomized, Parallel group | A Study to Evaluate the Efficacy and Safety of Remdesivir Plus Tocilizumab Compared With Remdesivir Plus Placebo in Hospitalized Participants With Severe COVID-19 Pneumonia | 53 centers, USA | Clinical Status as Assessed by the Investigator Using a 7-Category Ordinal Scale of Clinical Status on Day 28  Time to Clinical Improvement (TTCI) Defined as Time from Randomization to National Early Warning Score 2 (NEWS2) Score of </= 2 Maintained for 24 Hours Time to Improvement of at Least 2 Categories Relative to Baseline on a 7-Category Ordinal Scale of Clinical Status (…) | NCT04409262 |

1. **Supplementary Figure 1. Flow chart of the study**


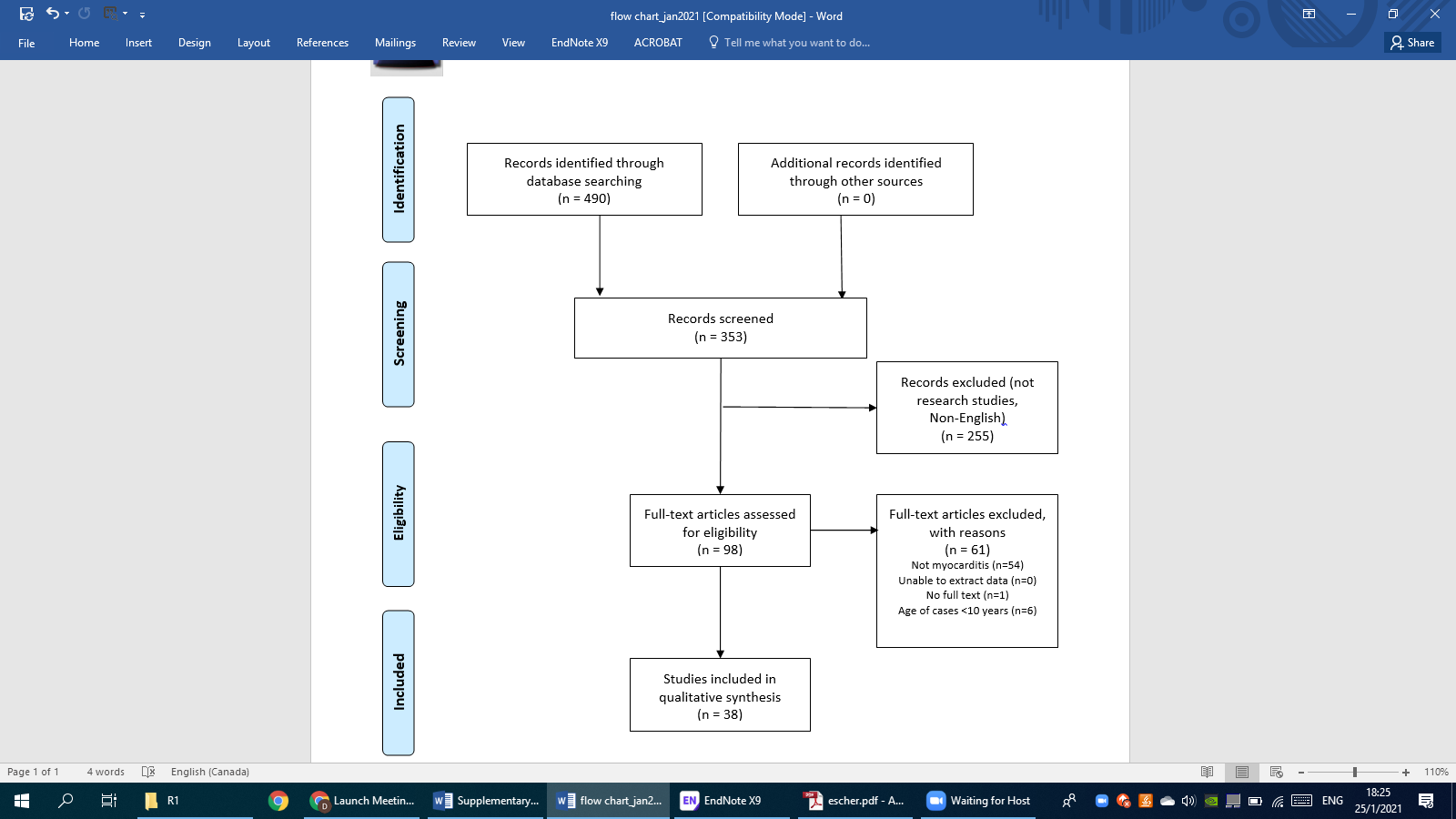


**SUPPLEMENTARY REFERENCES**

1. Caforio AL, Pankuweit S, Arbustini E, Basso C, Gimeno-Blanes J, Felix SB, Fu M, Heliö T, Heymans S, Jahns R, Klingel K, Linhart A, Maisch B, McKenna W, Mogensen J, Pinto YM, Ristic A, Schultheiss HP, Seggewiss H, Tavazzi L, Thiene G, Yilmaz A, Charron P, Elliott PM. Current state of knowledge on aetiology, diagnosis, management, and therapy of myocarditis: a position statement of the European Society of Cardiology Working Group on Myocardial and Pericardial Diseases. Eur Heart J 2013;**34**(33):2636-48, 2648a-2648d.

2. Friedrich MG, Sechtem U, Schulz-Menger J, Holmvang G, Alakija P, Cooper LT, White JA, Abdel-Aty H, Gutberlet M, Prasad S, Aletras A, Laissy JP, Paterson I, Filipchuk NG, Kumar A, Pauschinger M, Liu P. Cardiovascular magnetic resonance in myocarditis: A JACC White Paper. J Am Coll Cardiol 2009;**53**(17):1475-87.

3. Inciardi RM, Lupi L, Zaccone G, Italia L, Raffo M, Tomasoni D, Cani DS, Cerini M, Farina D, Gavazzi E, Maroldi R, Adamo M, Ammirati E, Sinagra G, Lombardi CM, Metra M. Cardiac Involvement in a Patient With Coronavirus Disease 2019 (COVID-19). JAMA Cardiol 2020.

4. Sala S, Peretto G, Gramegna M, Palmisano A, Villatore A, Vignale D, De Cobelli F, Tresoldi M, Cappelletti AM, Basso C, Godino C, Esposito A. Acute myocarditis presenting as a reverse Tako-Tsubo syndrome in a patient with SARS-CoV-2 respiratory infection. Eur Heart J 2020;**41**(19):1861-1862.

5. Doyen D, Moceri P, Ducreux D, Dellamonica J. Myocarditis in a patient with COVID-19: a cause of raised troponin and ECG changes. Lancet 2020;**395**(10235):1516.

6. Varga Z, Flammer AJ, Steiger P, Haberecker M, Andermatt R, Zinkernagel AS, Mehra MR, Schuepbach RA, Ruschitzka F, Moch H. Endothelial cell infection and endotheliitis in COVID-19. Lancet 2020;**395**(10234):1417-1418.

7. Luetkens JA, Isaak A, Zimmer S, Nattermann J, Sprinkart AM, Boesecke C, Rieke GJ, Zachoval C, Heine A, Velten M, Duerr GD. Diffuse Myocardial Inflammation in COVID-19 Associated Myocarditis Detected by Multiparametric Cardiac Magnetic Resonance Imaging. Circ Cardiovasc Imaging 2020;**13**(5):e010897.

8. Hu H, Ma F, Wei X, Fang Y. Coronavirus fulminant myocarditis treated with glucocorticoid and human immunoglobulin. European Heart Journal 2020.

9. Paul JF, Charles P, Richaud C, Caussin C, Diakov C. Myocarditis revealing COVID-19 infection in a young patient. Eur Heart J Cardiovasc Imaging 2020.

10. Coyle J, Igbinomwanhia E, Sanchez-Nadales A, Danciu S, Chu C, Shah N. A Recovered Case of COVID-19 Myocarditis and ARDS Treated with Corticosteroids, Tocilizumab, and Experimental AT-001. JACC Case Rep 2020.

11. Kim IC, Kim JY, Kim HA, Han S. COVID-19-related myocarditis in a 21-year-old female patient. Eur Heart J 2020;**41**(19):1859.

12. Craver R, Huber S, Sandomirsky M, McKenna D, Schieffelin J, Finger L. Fatal Eosinophilic Myocarditis in a Healthy 17-Year-Old Male with Severe Acute Respiratory Syndrome Coronavirus 2 (SARS-CoV-2c). Fetal Pediatr Pathol 2020:1-6.

13. Trogen B, Gonzalez FJ, Shust GF. COVID-19-Associated Myocarditis in an Adolescent. Pediatr Infect Dis J 2020;**39**(8):e204-e205.

14. Beşler MS, Arslan H. Acute myocarditis associated with COVID-19 infection. Am J Emerg Med 2020.

15. Gnecchi M, Moretti F, Bassi EM, Leonardi S, Totaro R, Perotti L, Zuccaro V, Perlini S, Preda L, Baldanti F, Bruno R, Visconti LO. Myocarditis in a 16-year-old boy positive for SARS-CoV-2. Lancet 2020;**395**(10242):e116.

16. Garot J, Amour J, Pezel T, Dermoch F, Messadaa K, Felten ML, Raymond V, Baubillier E, Sanguineti F, Garot P. SARS-CoV-2 Fulminant Myocarditis. JACC Case Rep 2020;**2**(9):1342-1346.

17. Al-Assaf O, Mirza M, Musa A. Atypical presentation of COVID-19 as subclinical myocarditis with persistent high degree Atrio-ventricular block treated with pacemaker implant. HeartRhythm Case Rep 2020.

18. Salamanca J, Díez-Villanueva P, Martínez P, Cecconi A, González de Marcos B, Reyes G, Salas C, Segovia J, Jiménez-Borreguero LJ, Alfonso F. COVID-19 "Fulminant Myocarditis" Successfully Treated With Temporary Mechanical Circulatory Support. JACC Cardiovasc Imaging 2020.

19. Bonnet M, Craighero F, Harbaoui B. Acute Myocarditis With Ventricular Noncompaction in a COVID-19 Patient. JACC Heart Fail 2020;**8**(7):599-600.

20. De Vita S, Ippolito S, Caracciolo MM, Barosi A. Peripartum cardiomyopathy in a COVID-19-infected woman: differential diagnosis with acute myocarditis-A case report from a Hub Institution during the COVID-19 outbreak. Echocardiography 2020.

21. Pavon AG, Meier D, Samim D, Rotzinger DC, Fournier S, Marquis P, Monney P, Muller O, Schwitter J. First Documentation of Persistent SARS-Cov-2 Infection Presenting With Late Acute Severe Myocarditis. Can J Cardiol 2020;**36**(8):1326.e5-1326.e7.

22. Ford JS, Holmes JF, Jones RF. Cardioembolic Stroke in a Patient with Coronavirus Disease of 2019 (COVID-19) Myocarditis: A Case Report. Clin Pract Cases Emerg Med 2020;**4**(3):332-335.

23. Richard I, Robinson B, Dawson A, Aya A, Ali R. An Atypical Presentation of Fulminant Myocarditis Secondary to COVID-19 Infection. Cureus 2020;**12**(7):e9179.

24. Warchoł I, Dębska-Kozłowska A, Karcz-Socha I, Książczyk M, Szymańska K, Lubiński A. Terra incognita: clinically suspected myocarditis in a patient with severe acute respiratory syndrome coronavirus 2 infection. Pol Arch Intern Med 2020;**130**(5):446-448.

25. Jacobs W, Lammens M, Kerckhofs A, Voets E, Van San E, Van Coillie S, Peleman C, Mergeay M, Sirimsi S, Matheeussen V, Jansens H, Baar I, Vanden Berghe T, Jorens PG. Fatal lymphocytic cardiac damage in coronavirus disease 2019 (COVID-19): autopsy reveals a ferroptosis signature. ESC Heart Fail 2020.

26. Hua A, O'Gallagher K, Sado D, Byrne J. Life-threatening cardiac tamponade complicating myo-pericarditis in COVID-19. Eur Heart J 2020;**41**(22):2130.

27. Cizgici AY, Zencirkiran Agus H, Yildiz M. COVID-19 myopericarditis: It should be kept in mind in today's conditions. Am J Emerg Med 2020;**38**(7):1547.e5-1547.e6.

28. Hussain H, Fadel A, Alwaeli H, Guardiola V. Coronavirus (COVID-19) Fulminant Myopericarditis and Acute Respiratory Distress Syndrome (ARDS) in a Middle-Aged Male Patient. Cureus 2020;**12**(6):e8808.

29. Spano G, Fischer K, Maillat C, Vicario G, Huber AT, Gräni C. Delayed isolated peri-myocarditis in a Covid-19 patient with respiratory symptoms but without lung involvement. Int J Cardiovasc Imaging 2020:1-2.

30. Dalen H, Holte E, Guldal AU, Hegvik JA, Stensaeth KH, Braaten AT, Mjølstad OC, Rossvoll O, Wiseth R. Acute perimyocarditis with cardiac tamponade in COVID-19 infection without respiratory disease. BMJ Case Rep 2020;**13**(8).

31. Labani A, Germain P, Douchet MP, Beghi M, Von Hunolstein JJ, Zeyons F, Roy C, El Ghannudi S. Acute Myopericarditis in a Patient With Mild SARS-CoV-2 Respiratory Infection. CJC Open 2020;**2**(5):435-437.

32. Khatri A, Wallach F. Coronavirus disease 2019 (Covid-19) presenting as purulent fulminant myopericarditis and cardiac tamponade: A case report and literature review. Heart Lung 2020.

33. Albert CL, Carmona-Rubio AE, Weiss AJ, Procop GG, Starling RC, Rodriguez ER. The Enemy Within: Sudden-Onset Reversible Cardiogenic Shock With Biopsy-Proven Cardiac Myocyte Infection by Severe Acute Respiratory Syndrome Coronavirus 2. Circulation 2020;**142**(19):1865-1870.

34. Nicol M, Cacoub L, Baudet M, Nahmani Y, Cacoub P, Cohen-Solal A, Henry P, Adle-Biassette H, Logeart D. Delayed acute myocarditis and COVID-19-related multisystem inflammatory syndrome. ESC Heart Fail 2020;**7**(6):4371-6.

35. Gauchotte G, Venard V, Segondy M, Cadoz C, Esposito-Fava A, Barraud D, Louis G. SARS-Cov-2 fulminant myocarditis: an autopsy and histopathological case study. Int J Legal Med 2021:1-5.

36. Caraffa R, Marcolongo R, Bottio T, Rizzo S, Bifulco O, Bagozzi L, D'Onofrio A, Caforio ALP, Jorgji V, Basso C, Gerosa G. Recurrent autoimmune myocarditis in a young woman during the coronavirus disease 2019 pandemic. ESC Heart Fail 2020.

37. Iqbal QZ, Haider MA, Sattar SBA, Hanif M, Javid I. COVID-19 Induced Myocarditis: A Rare Cause of Heart Failure. Cureus 2020;**12**(11):e11690.

38. Othenin-Girard A, Regamey J, Lamoth F, Horisberger A, Glampedakis E, Epiney JB, Kuntzer T, de Leval L, Carballares M, Hurni CA, Rusca M, Pantet O, Di Bernardo S, Oddo M, Comte D, Piquilloud L. Multisystem inflammatory syndrome with refractory cardiogenic shock due to acute myocarditis and mononeuritis multiplex after SARS-CoV-2 infection in an adult. Swiss Med Wkly 2020;**150**:w20387.

39. Escher F, Pietsch H, Aleshcheva G, Bock T, Baumeier C, Elsaesser A, Wenzel P, Hamm C, Westenfeld R, Schultheiss M, Gross U, Morawietz L, Schultheiss HP. Detection of viral SARS-CoV-2 genomes and histopathological changes in endomyocardial biopsies. ESC Heart Fail 2020;**7**(5):2440-2447.
